# Transcriptome Analysis of Circulating PBMCs to Understand Mechanism of High Altitude Adaptation in Native Cattle of Ladakh Region

**DOI:** 10.1038/s41598-018-25736-7

**Published:** 2018-05-16

**Authors:** Preeti Verma, Ankita Sharma, Monika Sodhi, Kiran Thakur, Ranjit S. Kataria, Saket K. Niranjan, Vijay K. Bharti, Prabhat Kumar, Arup Giri, Sahil Kalia, Manishi Mukesh

**Affiliations:** 1 Singhania University, Jhunjhunu, Rajasthan India; 2 ICAR-National Bureau of Animal Genetic Resources, Karnal, Haryana India; 3 Defence Institute of High Altitude Research, Leh, India

## Abstract

Ladakhi cattle is native population of Leh and Ladakh region and constantly exposed to hypobaric hypoxia over many generations. In present study, transcriptome signatures of cattle from Ladakh region (~5500 m) and Sahiwal cattle from tropical regions were evaluated using Agilent 44 K microarray chip. The top up-regulated genes in Ladakhi cows were *INHBC*, *ITPRI*, *HECA*, *ABI3*, *GPR*1*7*1, and *HIF*-1*α* involved in hypoxia and stress response. In Sahiwal cows, the top up-regulated genes *eEF1A1*, *GRO1*, *CXCL2*, *DEFB3* and *BOLA-DQA3* were associated with immune function and inflammatory response indicating their strong immune potential to combat the pathogens prevalent in the tropical conditions. The molecular pathways highly impacted were MAPK signaling, ETC, apoptosis, TLR signaling and NF- kB signaling pathway indicating signatures of adaptive evolution of these two cattle types in response to diverse environments. Further, qPCR analysis revealed increased expression of DEGs such as *HIF-1*, *EPAS-1, VEGFA, NOS2*, and *GLUT-1/SLC2A*1 in cattle types from high altitude suggesting their pivotal role in association with high altitude adaptation. Based on data generated, native cattle of Ladakh region was found to be genetically distinct from native cattle adapted to the tropical region of India.

## Introduction

The adaptation of livestock to high altitude hypoxic environment is important as they play an important role in sustaining the socio-economic condition of the local residents. It is a well-known fact that high-altitude adaptation is an evolutionary process in mammals with considerable physiological changes so as to make them survive and perform optimally at the extreme environmental condition. Conversely, ‘acclimatization’ is an immediate physiological response to changing environments. The term “high-altitude adaptation” can thus be described as irreversible, long-term physiological response associated with heritable, behavioural and genetic changes. One of the characteristic environmental feature of high altitude region is sustained hypobaric hypoxia as reflected by lower oxygen (O_2_) pressure that results in the insufficient supply of O_2_ to the tissues^1^. On the basis of O_2_ concentration level, hypoxia can be defined as moderate (5–8% O_2_) or anoxic (less than 1% O_2_)^2^, whereas; on the basis of time scale, hypoxia can be defined as acute (lasting for seconds to minutes) or chronic (lasting for hours to days)^3^. Low O_2_ pressure along with low temperature presents numerous physiological challenges to the animals living at high altitude.

Animals living under harsh conditions of high altitude have the unique ability to adapt themselves to chronic hypoxia and low ambient temperature in comparison to the animals living at sea level^4^, by undergoing modifications at physiological, molecular and cellular levels. They respond to hypoxia by maintaining O_2_ delivery through increased respiration rate, red blood cell mass and blood volume exhibiting increased blood O_2_ carrying capacity. They also possess greater ability to enhance O_2_ uptake and its delivery to tissues by suppressing their metabolic activity^3^, and/or enhancing flux capacity of the O_2_ transport^5^. Further, animals offset their O_2_ transport system in order to maintain the tissue O_2_ level for their growth, development and reproduction^6^. The high altitude animals also initiate several oxidative mitochondrial metabolism and signal transduction pathways which cascade into a series of biochemical and physiological alterations that enable the animal to survive in hypoxic condition. Amongst all the mammals, yak represents the best example of high altitude adaptation. They are genetically adapted to hypoxia, low ambient temperature, strong solar radiation and alpine environment^7^ through natural selection over millennia. Hypoxia in animals stimulates the expression of hypoxia inducible factor-1α (*HIF-1α*), a heterodimeric transcription factor which further regulates the transcription of several hypoxia related genes associated with several processes like erythropoiesis, angiogenesis, and glycolysis^8^. Prolonged hypoxia also leads to enhanced generation of reactive oxygen species (ROS), and lipid peroxidation. In such a situation, antioxidants such as superoxide dismutase (SOD), glutathione-peroxidase (GPX) and catalase (CAT) are activated. These antioxidants reduce the oxidative stress by preventing damage to important cellular components caused by ROS such as free radicals, peroxides, lipid peroxides and heavy metals^9^. Thus, high altitude environment presents numerous physiological challenges to the animal.

In India, one such region Leh and Ladakh also known as “land of high passes” is situated at an altitude of 3,500–5,500 m. It harbors difficult terrain, barren lands and little vegetation with cold arid and hypoxic conditions. This region in the state of Jammu and Kashmir lies between the Kunlun mountain range in the north and the main Great Himalayas to the south. The temperature here drops below −20 °C in winter season which lasts for about six months. Leh and Ladakh region is blessed with several native domesticated livestock species such as yak, yak and cattle cross (dzo, dzomo), mountain goats, sheep, cattle, horse, donkeys and double hump camel. Each of these species has developed an effective mechanism to survive at high altitude, particularly to combat the low temperature and low O_2_ condition. These animal genetic resources are very important in the life of the local people of Ladakh region as land resources are meagre and their livelihood is mainly dependent on these animals. Under such harsh climatic conditions, the survival and performance of exotic breeds is not a viable option as the lack of O_2_ and fodder/feeds at higher altitudes, only allow the well adapted animal genetic resources to thrive and perform.

The local cattle from Leh and Ladakh region is one such native population that has evolved naturally over the years and got adapted to the harsh high altitude hypoxia environment. This cattle is the lifeline of the local people and plays a pivotal role in fulfilling their nutritional needs. The subsistence on poor quality feed/fodder and better resistance capabilities to withstand environmental stress make this cattle distinct and preferred population by local people. Further, naturally evolved Ladakhi cattle could be a rich source of gene pool, as it might have inherited beneficial traits which allow them to combat the extreme environmental conditions.

Keeping in view the remarkable adaptive traits and importance, the Ladakhi population is an interesting resource to mine the genes and pathways associated with high altitude adaptation. In the present study, the transcriptome signature of peripheral blood mononuclear cells (PBMCs) of Ladakhi cows adapted to high altitude *vis* a *vis* Sahiwal cows adapted to the arid/semi-arid region at mean sea level was established using bovine expression microarray chips.

## Results

### Identification of DEGs in PBMCs of Ladakhi and Sahiwal cows

**Figure 1:**
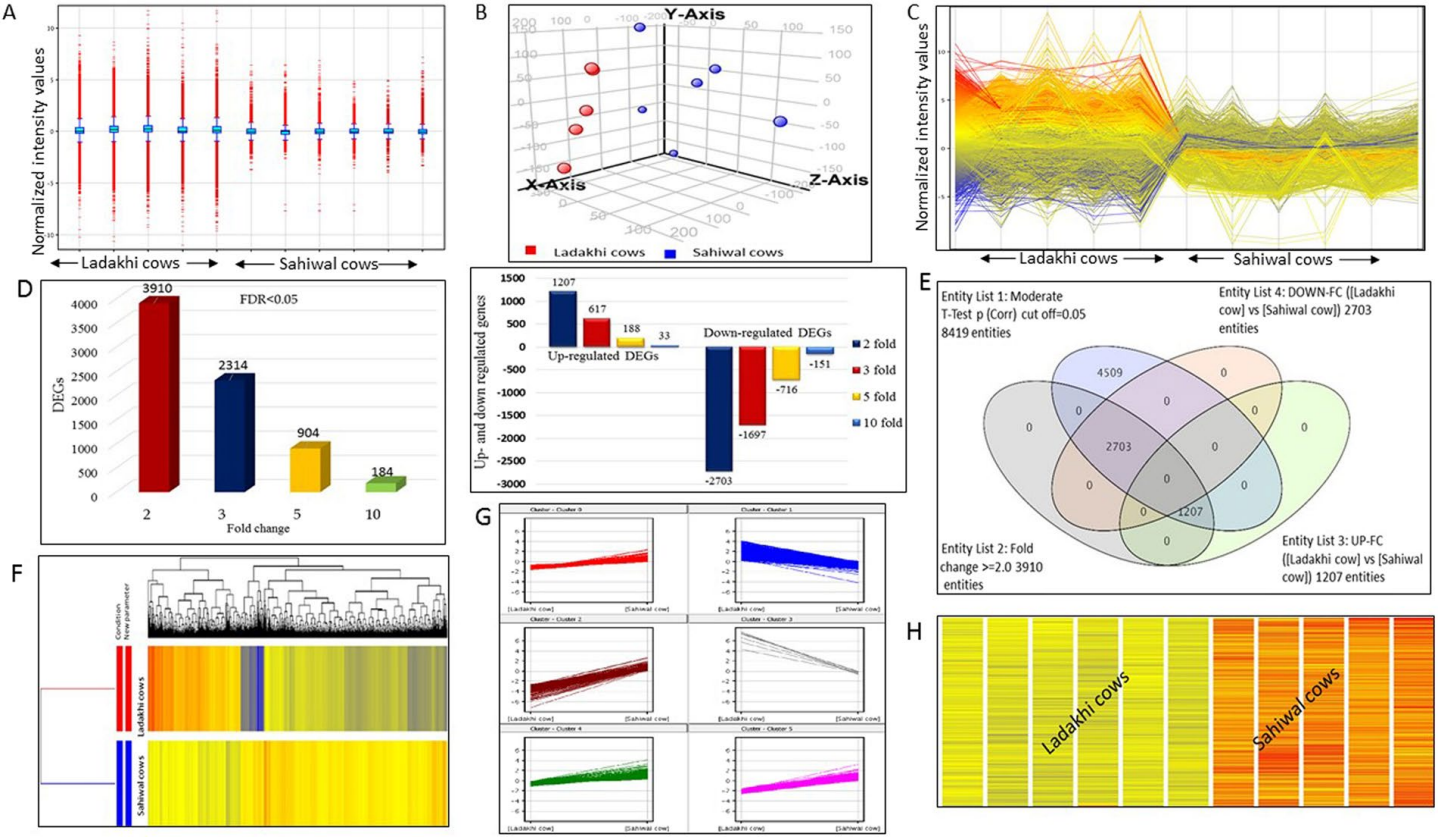
Identified DEGs in PBMCs of Ladakhi and Sahiwal Cows. (**A**) Box whisker plot showing distribution of normalized intensity values. (**B**) Principle component analysis (PCA) plot of Ladakhi and Sahiwal cows. (**C**) Line plot of DEGs between PBMCs of Ladakhi cows and Sahiwal cows at fold change criteria of ≥2. (**D**) Bar graph showing total number of DEGs and up-regulated and down-regulated genes between PBMCs of Ladakhi cows and Sahiwal cows at FDR < 0.05 and different fold change criteria’s. (**E**) Venn diagram showing number of up-regulated (2703) and down-regulated (1207) DEGs in PBMCs of Ladakhi cows. (**F**) Dendrogram view of hierarchical clustering with significant DEGs in PBMCs of Ladakhi and Sahiwal cows. (**G**) Normalized expression profile of DEGs in PBMCs of Ladakhi cows at 2 fold change identified by k-means clustering. (**H**) Heat map view of DEGs in PBMCs of Ladakhi and Sahiwal cows.

In the present study, the transcriptome profile of PBMCs of Ladakhi cows native to Leh and Ladakh region (high altitude) was generated. To understand the transcriptomic variation caused by difference in altitude, transcriptome profile of PBMCs of Sahiwal cows adapted to tropical condition (mean sea level) was also generated. The transcriptome data was generated using bovine gene expression microarray from Agilent Technologies (Product number: G2519F). The Agilent bovine oligonucleotide array used in this study was in 4 × 44 K format with design ID V2:023647 and 43,803 probes. The extracted RNA was intact and sufficiently good in quality as indicated by electropherogram with two characteristics peaks of 18S rRNA & 28S rRNA. (Supplementary Fig. S1). To ensure successful microarray hybridization, the yield and specific activity of Cyanine3 (Cy3)-labeled complementary RNA (cRNA) was measured in terms of Cy3 dye concentration (pmol/μl), RNA absorbance ratio (260 nm/280 nm) and cRNA concentration (ng/μl). The concentration of cRNA (ng/μl) was used to determine the μg cRNA yield and concentrations of cRNA (ng/μl) and Cy3 (pmol/μl) was used to determine the specific activity. In all the samples of Ladakhi cows and Sahiwal cows, the yield of cRNA was >1.65 μg and specific activity was >9.0 pmol Cy3 per μg cRNA, indicating the good quality of cRNA obtained. The scanned images analysed using Agilent feature extraction software showed successful hybridization of the cRNA samples. The microarray raw data files were subjected for quantile normalization to remove unwanted technical variation. Based on intensity distribution, the box whisker plot showed a consistent distribution of normalized intensity values in samples of Ladakhi and Sahiwal cows (Fig. 1A). In addition principle component analysis plot (Fig. 1B) revealed distinct grouping of Ladakhi and Sahiwal cows based on transcriptome data. The normalized transcriptome data was filtered based on multiple testing corrected P values, P < 0.05 (FDR < 0.05) and different fold change criteria. Line plot of transcripts showing differential genes expression of between Ladakhi and Sahiwal cows PBMCs is shown in Fig. 1C.

Overall microarray data analysis revealed a total of 8417 differentially expressed genes (DEGs) between Ladakhi and Sahiwal PBMCs with multiple testing (FDR < 0.05; Fig. 1D). With additional cut off criteria *i.e*., fold change of 2 or more, a total of 3910 genes were found to be differentially expressed between the two cattle types. Venn analysis showed that out of these 3910 genes, 1207 were up-regulated while 2703 genes were down-regulated in Ladakhi cows in comparison to Sahiwal cows (Fig. 1E). Overall analysis revealed substantial differences in transcriptome signature between the two cattle types.

### Clustering of transcriptome data

The normalized transcriptome data was partitioned using hierarchical and *k*-means clustering tools. For creating a hierarchical clustering, a total of 3910 DEGs were used (Fig. 1F). The *k-*mean clustering yielded a total of 6 major clusters (Fig. 1G). These clusters helped to differentiate the genes on the basis of their expression pattern between Ladakhi and Sahiwal cows. The heat maps thus generated helped to judge the similarities/patterns between genes and between samples. The clustering tools and heat map collectively revealed distinct transcriptome signature for high altitude adapted Ladakhi cows and tropically adapted Sahiwal cows (Fig. 1H).

### Top up-regulated DEGs in PBMCs of Ladakhi cows

Out of 3910 DEGs, a total of 1207 genes were found to be up-regulated in Ladakhi cows. List of top 50 DEGs with their absolute fold change values (Fc), log fold change values (Log Fc), gene symbol, gene description are presented in Table 1.

**Table 1:** List of top 50 up-regulated genes in PBMCs of Ladakhi cows (FDR < 0.05, Fold change ≥2-or ≤2).

**Oligo ID**	**Gene Symbol**	**p (Corr)**	**Fc (abs)**	**Log Fc**	**Description**
A_73_119248	*INHBC*	1.15E-07	197.63	7.63	Inhibin, beta C
A_73_P063316	*ITPR1*	1.15E-07	75.38	6.24	Inositol 1,4,5-trisphosphate receptor, type 1
A_73_P081586	*HECA*	5.94E-06	58.35	5.87	Headcase homolog (Drosophila)
A_73_P092856	*VAV3*	4.24E-05	18.98	4.25	Guanine nucleotide exchange factor
A_73_115177	*ARNT2*	4.22E-05	18.89	4.23	Aryl-hydrocarbon receptor nuclear translocator 2
A_73_P446651	*HIF3A*	4.20E-05	18.53	4.21	Hypoxia inducible factor 3
A_73_P392791	*NOS2*	3.24E-05	17.46	4.13	Nitric oxide synthase 2
A_73_P038606	*HIF1A*	4.24E-05	15.66	3.97	Hypoxia inducible factor 1, alpha subunit inhibitor
A_73_P061311	*PAG11*	0.001017	15.46	3.95	Pregnancy-associated glycoprotein 11
A_73_P084276	*C7H19orf80*	3.54E-08	14.90	3.90	Chromosome 19 open reading frame 80
A_73_104606	*ABI3*	9.50E-08	13.88	3.80	ABI family, member 3
A_73_104104	*GPR171*	1.85E-05	13.41	3.75	G protein-coupled receptor 171
A_73_P333276	*SLC2A3*	3.22E-08	12.21	3.61	Solute carrier family 3
A_73_106569	*LOC100848734*	7.51E-04	12.15	3.60	Histone H2A type 1-D-like
A_73_P102846	*CEBPA*	4.91E-06	12.05	3.59	CCAAT/enhancer binding protein (C/EBP), alpha
A_73_108614	*TRPM4*	1.96E-05	11.74	3.55	Transient receptor potential cation channel, subfamily M, member 4
A_73_P086716	*SLC28A3*	0.008893	11.37	3.51	Solute carrier family 28, member 3
A_73_P485720	*LOC100851189*	2.24E-06	10.57	3.40	40S ribosomal protein s15a-like
A_73_P046856	*SPINLW1*	0.003058	10.39	3.38	Serine peptidase inhibitor-like, with Kunitz and WAP domains 1
A_73_110087	*ARL6*	3.43E-06	10.22	3.35	ADP-ribosylation factor-like 6
A_73_100057	*SLAMF7*	1.49E-05	9.51	3.25	SLAM family member 7
A_73_P044771	*NEFL*	1.16E-04	9.48	3.24	Neurofilament, light polypeptide
A_73_P445731	*TDRD6*	6.76E-04	9.20	3.20	Tudor domain containing 6
A_73_P047851	*ZNF215*	0.005834	9.07	3.18	Zinc finger protein 215
A_73_P132876	*PGAP1*	0.001281	9.01	3.17	Post-GPI attachment to proteins 1
A_73_100733	*ND4L*	1.50E-05	8.91	3.16	NADH-ubiquinone oxidoreductase chain 4L
A_73_P421611	*LRP4*	4.64E-06	8.19	3.03	Low density lipoprotein receptor-related protein 4
A_73_107115	*CCDC40*	5.85E-05	8.05	3.01	S coiled-coil domain containing 40
A_73_105601	*SLC34A2*	0.008773	8.04	3.01	Solute carrier family 34, member 2
A_73_112076	*CCL26*	2.02E-05	7.96	2.99	chemokine (C-C motif) ligand 26
A_73_P115046	*PCDHB1*	0.004305	7.94	2.99	Protocadherin beta 1
A_73_117690	*LPGAT1*	0.004075	7.92	2.99	Lysophosphatidylglycerol acyltransferase 1
A_73_P381221	*RBL1*	7.21E-04	7.73	2.95	Retinoblastoma-like 1
A_73_100280	*NPL*	3.68E-06	7.67	2.94	N-acetylneuraminate pyruvate lyase
A_73_110748	*NAV3*	1.20E-05	7.61	2.93	Neuron navigator 3
A_73_107697	*SMEK2*	3.64E-04	7.46	2.90	SMEK homolog 2, suppressor of mek1 (Dictyostelium)
A_73_P206097	*LCOR*	1.83E-05	7.42	2.89	Ligand dependent nuclear receptor corepressor
A_73_P258781	*CIDEC*	3.27E-04	7.35	2.88	Cell death-inducing DFFA-like effector c
A_73_102403	*EFNB3*	0.045754	7.28	2.86	Ephrin-B3
A_73_P063761	*GHRL*	1.43E-04	7.09	2.83	Ghrelin/obestatin prepropeptide
A_73_118319	*VEGFC*	1.20E-05	6.93	2.79	Vascular endothelial growth factor C
A_73_P1544174	*SCNM1*	1.19E-05	6.90	2.79	Sodium channel modifier 1
A_73_116472	*TAB2*	3.70E-04	6.86	2.78	TGF-beta activated kinase 1/MAP3K7 binding protein 2
A_73_P331481	*KRT80*	0.002596	6.79	2.76	Keratin 80
A_73_P101446	*CACNG2*	1.90E-04	6.74	2.75	Calcium channel, voltage-dependent, gamma subunit 2
A_73_P349241	*TXNDC8*	1.22E-04	6.70	2.74	Thioredoxin domain containing 8
A_73_119350	*HTR7*	0.023272	6.69	2.74	5-hydroxytryptamine receptor 7
A_73_P043406	*NEPN*	6.40E-07	6.69	2.74	S nephrocan
A_73_P104121	*SENP2*	6.38E-07	6.63	2.72	Sentrin/SMT3 specific peptidase 2

Some of the top most up-regulated genes in Ladakhi cows were; Inhibin, beta C (*INHBC*), Inositol 1,4,5-trisphosphate receptor, type 1 (*ITPRI*), Headcase homolog (*HECA*), ABI family, member 3 (*ABI3*), G protein-coupled receptor 171 (*GPR171*), Cytochrome P450, subfamily 1 (*CYPIAI*), Solute carrier family 28 (*SLC283*), Serine peptidase inhibitor-like, with Kunitz and WAP domains 1 (*SPINLW1*), ADP-ribosylation factor-like 6 (*ARL6*), hypoxia inducing factor-alpha (*HIF1A*), hypoxia inducing factor 3A (*HIF3A*), vascular endothelial growth factor-A (*VEGFA*), nitric oxide synthase (*NOS2*), myosin heavy chain 2 (*MYH2*), matrix metalloproteinases 1 (*MMP1*), mucin 1 cell surface associated (*MUC1*), apelin (*APLN*), fizzled family receptor 3 (*FZD3*), adenosine monophosphate-activated protein kinase (*PRKAA1*) and sentrin-specific peptidase 2 (*SENP2*) *etc*. Some of the already known high altitude adaptation candidate genes induced under hypoxia environment were also found to be up-regulated in Ladakhi cows (Table 2). For example, the hypoxia inducing factors *HIF-1α* and *HIF3A* were up-regulated by 15.66 and 18.53 folds, respectively in Ladakhi cows. Endothelial PAS Domain1 (*EPAS-1*) was upregulated 19.72 fold in Ladakhi cows. The nitric oxide synthase (*NOS2*) was up-regulated by 17.46 fold and substantiated the previous studies that high altitude hypoxia increases the expression of inducible nitric oxide synthase. The calcium channel, voltage-dependent, T type, alpha 1G subunit (*CACNA1G*) was upregulated by 26.51 fold change in Ladakhi cattle. Vascular endothelial growth factors; *VEGFA, VEGFB* and *VEGFC* were also found to be up-regulated by 6.35, 4.75 and 6.93 folds, respectively. Myosin heavy chain 2 (*MYH2*) gene related to skeletal muscle contraction and regulates processes like glycolysis and glucose metabolism was also up-regulated by 2.83 fold. The hexokinase 2 (*HK2*) gene involved in anaerobic metabolism was also observed to be up-regulated 8.01 fold in Ladakhi as compared to Sahiwal cows. The higher expression of this gene suggested increased glucose metabolism under hypoxic environment. In addition, several genes known to be hypoxia-responsive namely, matrix metalloproteinases 1 (*MMP1*, 2.45 fold), mucin 1 cell surface associated (*MUC1*, 3.82 fold), apelin (*APLN*, 19.98 fold), fizzled family receptor 3 (*FZD3*, 19.94 fold) were also observed to be upregulated. Amongst these, *APLN* and *FZD3* encode for factors having proangiogenic functions as reported in different models under hypoxic conditions. Another important gene up-regulated 5.29 fold in Ladakhi cows was α-1 catalytic subunit of adenosine monophosphate-activated protein kinase (*PRKAA1*, also known as *AMPKα1*). Furthermore, sentrin-specific peptidase 2 (*SENP2*), an enzyme implicated in erythropoiesis was also found to be up-regulated (6.63 fold) in Ladakhi cows. Other interesting genes showing upregulation in Ladakhi cows were mitochondrial calcium uptake 1 (*MICU1*, 11.10 fold), vav 3 guanine nucleotide exchange factor (*VAV3*, 18.98 fold), vav 2 guanine nucleotide exchange factor (*VAV2*, 13.79 fold), and aryl-hydrocarbon receptor nuclear translocator 2 (*ARNT2*, 18.89 fold).

**Table 2:** List of up-regulated candidate genes in PBMCs of Ladakhi cows (hypoxia related) and Sahiwal cows (immune and stress related genes).

Oligo ID	Gene symbol	Fc (abs)	Log Fc	Description
**Ladakhi cows**				
A_73_118958	*CACNA1G*	26.51	4.73	calcium channel, voltage-dependent, T type, alpha 1G subunit
A_73_121361	*NOX-1*	20.97	4.39	NADPH oxidase 1
A_73_P323491	*APLN*	19.98	4.32	Apelin
A_73_P085882	*FZD3*	19.94	4.32	frizzled family receptor 3
A_73_P033606	*EPAS-1*	19.72	4.30	Endothelial PAS Domain1
A_73_P032076	*CASQ2*	19.37	4.27	Calsequestrin 2 (cardiac muscle)
A_73_P092856	*VAV3*	18.98	4.25	Guanine nucleotide exchange factor
A_73_115177	*ARNT2*	18.89	4.23	Aryl-hydrocarbon receptor nuclear translocator 2
A_73_P446651	*HIF3A*	18.53	4.21	Hypoxia inducible factor 3
A_73_P346251	*SLC2A1*	17.78	4.15	Solute carrier family 2
A_73_P107246	*RTEL1*	17.53	4.13	Regulator of telomere elongation helicase 1
A_73_P392791	*NOS2*	17.46	4.13	Nitric oxide synthase 2
A_73_111390	*ACE*	16.83	4.07	Angiotensin I converting enzyme
A_73_P038606	*HIF1A*	15.66	3.97	Hypoxia inducible factor 1, alpha subunit inhibitor
A_73_P091611	*FOXM1*	14.47	3.85	Forkhead box M1
A_73_P106891	*VAV2*	13.79	3.78	Vav 2 guanine nucleotide exchange factor
A_73_P333276	*SLC2A3*	12.21	3.61	Solute carrier family 3
A_73_101701	*BRCC3*	11.88	3.57	BRCA1/BRCA2-containing complex, subunit 3
A_73_P256461	*MICU1*	11.10	3.45	Mitochondrial calcium uptake 1
A_73_P327811	*ACSS3*	9.10	3.18	Acyl-CoA synthetase short-chain family member 3
A_73_P082152	*HK2*	8.01	3.00	Hexokinase 2
A_73_118319	*VEGFC*	6.93	2.79	Vascular endothelial growth factor C
A_73_P104121	*SENP2*	6.63	2.72	Sentrin specific peptidase 2
A_73_P108076	*VEGFA*	6.35	2.67	Vascular endothelial growth factor A
A_73_P069981	*PRKAA1*	5.29	2.40	Protein kinase, AMP-activated, alpha 1 catalytic subunit
A_73_P061496	*VEGFB*	4.75	2.25	Vascular endothelial growth factor B
A_73_P397951	*MUC1*	3.82	1.93	Mucin 1, cell surface associated
A_73_P323491	*MYH2*	2.83	1.50	Myosin, heavy chain 2, skeletal muscle
A_73_100846	*MMP1*	2.45	1.29	Matrix metallopeptidase 1
A_73_P387351	*SIRT7*	2.27	1.18	Sirtuin 7
**Sahiwal cows**				
**Immune genes**				
A_73_P042021	*GRO1*	53.93	5.75	Chemokine (C-X-C motif) ligand 1
A_73_114724	*CXCL2*	42.50	5.41	Chemokine (C-X-C motif) ligand 2
A_73_P054346	*DEFB3*	30.42	4.93	Defensin beta 3
A_73_108688	*CXCL3*	29.06	4.86	Chemokine (C-X-C motif) ligand 2
A_73_118124	*BOLA-DQA3*	23.13	4.53	Major histocompatibility complex, class II, DQ alpha, type 3
A_73_P102796	*CCL4*	17.58	4.14	Chemokine (C-C motif) ligand 4
A_73_P064756	*CCL16*	13.90	3.80	Chemokine (C-C motif) ligand 16
A_73_P107936	*LA-DQB*	10.94	3.45	Major histocompatibility complex cell surface glycoprotein
A_73_110556	*IL1B*	10.80	3.43	Interleukin 1, beta
A_73_120710	*DEFB1*	8.56	3.10	Defensin, beta 1
A_73_P108361	*BOLA*	7.34	2.88	Major histocompatibility complex, class I, A
A_73_P031116	*DEFB4A*	7.12	2.83	Defensin, beta 4A
A_73_P031106	*DEFB10*	6.36	2.67	Defensin 10
A_73_P036601	*BOLA-DQA1*	4.94	2.30	Major histocompatibility complex, class II, DQ alpha, type 1
A_73_P052096	*BOLA-DQB*	4.12	2.04	Major histocompatibility complex, class II, DQ beta
A_73_P337156	*CX3CR1*	3.23	1.69	Chemokine (C-X3-C motif) receptor 1
A_73_P166557	*IL32*	3.18	1.67	Interleukin 32
A_73_P443111	*ILF2*	3.16	1.66	Interleukin enhancer binding factor 2, 45kda
A_73_P467773	*CCR7*	2.85	1.51	Chemokine (C-C motif) receptor 7
A_73_111391	*IL18*	2.28	1.19	Interleukin 18
A_73_108982	*IL4I1*	2.27	1.18	Interleukin 4 induced 1
A_73_P062206	*BOLA-DRB3*	2.22	2.22	Major histocompatibility complex, class II, DRB3
**Oxidative and heat stress genes**				
A_73_121452	*DNAJC2*	21.66	4.44	Dnaj (Hsp40) homolog, subfamily C, member 2
A_73_P100016	*SOD2*	7.98	3.00	Superoxide dismutase 2, mitochondrial
A_73_109073	*DNAJC27*	7.27	2.86	Dnaj (Hsp40) homolog, subfamily C, member 27
A_73_P046076	*GLRX2*	4.77	2.25	Glutaredoxin 2
A_73_116548	*HMOX1*	4.38	2.13	Heme oxygenase
A_73_120225	*HSPA4*	4.15	2.05	Heat shock 70kda protein 4
A_73_103760	*GPX7*	3.62	1.86	Gutathione peroxidase 7

### Top up-regulated DEGs in PBMCs of Sahiwal cows

A total of 2703 genes were up-regulated in PBMCs of tropically adapted Sahiwal cows in comparison to Ladakhi cows PBMCs. Some of the top most up-regulated genes in PBMCS of Sahiwal cow were: elongation factor 1-alpha 1 (*eEF1A1*), chemokine (C-X-C motif) ligand 1 (*GRO1*), chemokine (C-X-C motif) ligand 2 (*CXCL2*), colony stimulating factor 3 receptor (*CSF3R)*, defensin beta 3 (*DEFB3*), transforming growth factor, beta-induced (*TGFB3*), and major histocompatibility complex class II DQ alpha type 3 (*BOLA-DQA3*). List of genes with their absolute fold change values (Fc), log fold change values (Log Fc), gene symbol, gene description are presented in Table 3.

**Table 3:** List of top 50 up-regulated genes in PBMCs of Sahiwal cows (FDR < 0.05, Fold change ≥2).

Oligio ID	Gene Symbol	p (Corr)	Fc (abs)	Log Fc	Description
A_73_P492178	*eEF1A1*	0.0033	84.96	6.41	Elongation factor 1-alpha 1 - Homo sapiens
A_73_P042021	*GRO1*	6.77E-04	53.93	5.75	chemokine (C-X-C motif) ligand 1
A_73_114724	*CXCL2*	0.0035	42.50	5.41	chemokine (C-X-C motif) ligand 2
A_73_P490513	*LOC613345*	1.48E-04	38.71	5.27	H3 histone, family 3A-like
A_73_P093746	*CSF3R*	0.0020	38.13	5.25	Colony stimulating factor 3 receptor
A_73_P054346	*DEFB3*	6.02E-04	30.42	4.93	Defensin beta 3
A_73_108688	*CXCL3*	0.0128	29.06	4.86	chemokine (C-X-C motif) ligand 2
A_73_P042156	*NSMCE4A*	3.00E-06	28.26	4.82	Non-SMC element 4 homolog A (S. Cerevisiae)
A_73_112587	*TGFBI*	0.0021	26.79	4.74	Transforming growth factor, beta-induced
A_73_118124	*BOLA-DQA3*	0.0034	23.13	4.53	Major histocompatibility complex, class II, DQ alpha, type 3
A_73_P091851	*LOC786131*	1.11E-05	22.61	4.50	Ribosomal protein L17-like
A_73_106226	*BTK*	8.78E-04	22.48	4.49	Bruton agammaglobulinemia tyrosine kinase
A_73_P112801	*SMIM4*	1.64E-04	22.08	4.46	Small integral membrane protein 4
A_73_121452	*DNAJC2*	1.35E-05	21.66	4.44	Dnaj (Hsp40) homolog, subfamily C, member 2
A_73_P095766	*URB1*	2.07E-05	21.26	4.41	URB1 ribosome biogenesis 1 homolog (S. Cerevisiae)
A_73_102402	*ICAM3*	0.0089	19.81	4.31	Intercellular adhesion molecule 3
A_73_100827	*MARVELD1*	4.24E-05	19.08	4.25	MARVEL domain containing 1
A_73_P050621	*TIMM8B*	7.17E-07	19.01	4.25	Translocase of inner mitochondrial membrane 8 homolog B
A_73_P461976	*MYC*	7.63E-04	17.96	4.17	V-myc myelocytomatosis viral oncogene homolog
A_73_P102796	*CCL4*	0.0319	17.58	4.14	chemokine (C-C motif) ligand 4
A_73_115248	*SURF6*	1.09E-06	17.28	4.11	Surfeit 6
A_73_110287	*SFT2D1*	0.0014	17.20	4.10	SFT2 domain containing 1
A_73_112071	*C25H16orf59*	0.0055	17.15	4.10	Chromosome 25 open reading frame, human
A_73_P045161	*YTHDF2*	5.99E-05	16.98	4.09	YTH domain family, member 2
A_73_P250966	*SLC46A2*	5.89E-05	16.77	4.07	Solute carrier family 46, member 2
A_73_101963	*BNIP3L*	0.0067	15.53	3.96	BCL2/adenovirus E1B 19kda interacting protein 3-like
A_73_P335761	*DDX47*	2.10E-04	15.46	3.95	DEAD (Asp-Glu-Ala-Asp) box polypeptide 47
A_73_115219	*ARHGEF3*	0.0021	14.82	3.89	Rho guanine nucleotide exchange factor
A_73_P255701	*ELAC1*	3.11E-05	14.74	3.88	Elac homolog 1 (E. Coli)
A_73_104190	*HP*	2.76E-04	14.67	3.87	Haptoglobin
A_73_117230	*GPR37L1*	2.63E-05	14.62	3.87	G protein-coupled receptor 37 like 1
A_73_P435496	*SDE2*	8.44E-05	14.14	3.82	SDE2 telomere maintenance homolog
A_73_P122936	*CALM*	1.40E-04	14.10	3.82	Calmodulin-like
A_73_112287	*SLBP*	0.008642	14.00	3.81	Stem-loop binding protein
A_73_109126	*MITD1*	9.44E-07	13.99	3.81	Microtubule interacting and transport, domain containing 1
A_73_P064756	*CCL16*	0.0046	13.90	3.80	chemokine (C-C motif) ligand 16
A_73_P044251	*NDRG1*	0.0159	13.79	3.79	N-myc downstream regulated 1
A_73_P312076	*RNF149*	0.0025	13.78	3.78	Ring finger protein 149
A_73_P119681	*SAT1*	0.0012	13.62	3.77	Spermidine/spermine N1-acetyltransferase 1
A_73_117315	*RTF1*	0.0022	13.46	3.75	Rtf1, Paf1/RNA polymerase II complex component, homolog
A_73_P043126	*CHCHD4*	0.003424	13.39	3.74	Coiled-coil-helix-coiled-coil-helix domain containing 4
A_73_117804	*COX17*	0.0091	13.35	3.74	COX17 cytochrome c oxidase assembly homolog (S. Cerevisiae
A_73_105727	*FAS*	7.21E-04	13.24	3.73	Fas (TNF receptor superfamily, member 6)
A_73_P030531	*ALKBH7*	0.0056	13.10	3.71	Alkb, alkylation repair homolog 7 (*E. Coli*
A_73_P089166	*LOC101909443*	7.41E-04	12.75	3.67	PREDICTED dynein light chain 1, cytoplasmic-like
A_73_100589	*IRF5*	2.49E-04	12.68	3.66	Interferon regulatory factor 5
A_73_P087251	*SEC24B*	1.23E-07	12.63	3.66	SEC24 family member B
A_73_P054601	*HEATR1*	0.0010	12.61	3.66	HEAT repeat containing protein 1
A_73_P037801	*LOC515418*	0.012048	12.57	3.65	CD1a molecule-like
A_73_114567	*GNL2*	0.002198	12.27	3.62	Guanine nucleotide binding protein-like 2 (nucleolar)

In Sahiwal cows, *eEF1A1* was found to be the top most up-regulated gene (84.96 fold) followed by *GRO1* (53.93 fold) and *CXCL2* (42.50 fold). Several other immune related genes like chemokine (C-C motif) ligand 3 (*CXCL3*, 29.06 fold), chemokine (C-C motif) ligand 4 (*CCL4*, 17.58 fold), chemokine (C-C motif) ligand 16 (*CCL16*, 13.90 fold), and colony stimulating factor 3 receptor were also present several folds higher in Sahiwal PBMCs. *CSF3R* was another major immune related gene that was up-regulated (38.13 fold) in Sahiwal PBMCs. Other important immune related genes upregulated in Sahiwal PBMCs were *BOLA DQA3* (23.13 fold), *BoLA* (7.34 fold), *BOLA DQA1* (4.94 fold), *BOLA DQB* (4.12 fold) and *BOLA DRB3* (2.22 fold). Up-regulation of beta defensing genes *viz*., *DEFB3* (30.42 fold), *DEFB1* (8.56 fold), *DEFB4A* (7.12 fold) and *DEFB10* (6.36 fold) in our data set was also noteworthy. List of some important immune and stress related genes upregulated in Sahiwal PBMCs is mentioned in Table 2.

The transforming growth factor TGF-beta (*TGFBI)* gene that is a part of transforming growth factor signaling pathway and is involved in many cellular processes including cell growth, differentiation and apoptosis, cellular homeostasis was also up-regulated (26.79 fold) in Sahiwal PBMCs. Apart from these genes, several genes of heat shock family showing up-regulation in Sahiwal PBMCs included *DNAJC2* (21.66 fold), *DNAJC27* (7.27 fold), *HSPA4* (4.15 fold), *HSPB11* (3.30 fold). The overexpression of cluster of genes related to DEAD Box polypeptides (*DDX*); *DDX47* (15.46 fold), *DDX55* (6.21 fold), *DDX6* (5.16 fold), *DDX18* (6.55 fold), *DDX21* (3.66 fold), *DDX23* (2.45 fold), *DDX54* (3.10 fold), *DDX59* (9.07 fold) were also significantly high in Sahiwal cows PBMCs. In addition, several ribosomal protein like genes *viz*; *RPL34* (5.29 fold), *RPL36* (2.72 fold), *RPS12* (1.36 fold), *RPS24* (2.68 fold), *MRPS9* (2.28 fold), *MRPS15* (2.53 fold), *MRPL44* (4.55 fold), *MRPL46 (*5.39 fold), *MRPS17* (4.44 fold), *MRPS30* (2.53 fold), *MRPL20* (4.87 fold), *MRPL34* (3.67 fold), *MRPL12* (3.50 fold), were significantly higher in Sahiwal cows.

Another category of genes that showed upregulation in Sahiwal was PHD finger protein genes including, PHF2 (2.83 fold), *PHF8* (2.03 fold), *PHF17* (4.45 fold) and *PHF20* (6.75 fold). These genes are related to methyl lysine-binding protein, a component of the MOF histone acetyltransferase protein complex. The higher expression of these genes might be attributed to the presence of higher atmospheric O_2_ that is must for their activity. Under high O_2_ content, PHD enzymes help in proline hydroxylation of *HIF-α* which is prerequisite for interaction of *HIF-α* with von Hippel Lindau (VHL) protein. This results in *HIF-α* degradation by proteosome under high O_2_ tension^10^. Other set of genes upregulated in Sahiwal included coagulation factor XIII A1 polypeptide (*F13A1*, 3.33 fold), the last zymogen to become activated in the blood coagulation cascade; elaC homolog1 (*ELAC1*, 14.74 fold) protein involved in RNA transport; cytochrome P450 family 27 (*CYP27A1*, 7.93 fold) instructing production of an enzyme called sterol 27-hydroxylase that plays a key role in maintaining normal cholesterol levels in the body; hemopoietic cell kinase (*HCK*, 4.64 fold) protein-tyrosine kinase that is predominantly expressed in hemopoietic cell types and may play a role in neutrophil migration; solute carrier family 46 member 2 (*SLC46A2*, 16.77 fold) with role in T cell differentiation, T cell homeostasis and thymus development; TBC1 domain family member 8 (*TBC1D8*, 6.55 fold) with important role in cell cycle, TBC1 domain family member 15 (*TBC1D15*, 2.26 fold); tubulin folding cofactor E (TBE) calmodulin-like (*CALM*, 14.10 fold)-calcium binding protein; adenosyl homocysteinase (*AHCY*, 2.69 fold)- regulating the intracellular S-adenosyl homocysteine concentration that is important for transmethylation reactions; Rtf1, Paf1/RNA polymerase II complex component (*RTF1*, 13.46 fold) involved in regulation of transcription elongation and chromatin remodeling; spermidine/spermine N1-acetyltransferase 1 (*SAT1*, 13.62 fold) that catalyzes the acetylation of spermidine, spermine, and also regulates intracellular concentration of polyamines and their transport out of cells.

### Gene ontology

**Figure 2:**
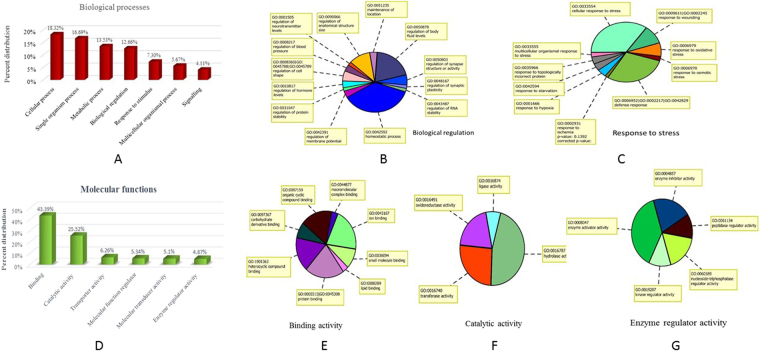
GO analysis of up-regulated genes in Ladakhi cows. (**A**) Enriched biological processes in Ladakhi cows. (**B**) Enriched biological processes under category “Biological regulation” in Ladakhi cows. (**C**) Enriched biological processes under sub category “Response to stress” in Ladakhi cows. (**D**) Enriched molecular functions in Ladakhi cows. (**E**) Enriched molecular functions under category “binding activity” in Ladakhi cows. (**F**) Enriched molecular functions under category “catalytic activity” in Ladakhi cows. (**G**) Enriched molecular functions under category “enzyme regulator activity” in Ladakhi cows.

The upregulated genes in Ladakhi and Sahiwal cows were further classified into classes depending upon their functional classes. The top biological process parental ontology (GO) terms identified in Ladakhi cows (Fig. 2A) included cellular process (GO: 0009987; p value: 0.2260), single organism process (GO: 0044699; p value: 0.0320), metabolic process (GO: 0008152; p value: 0.7966), biological regulation (GO: 0065007; p value: 0.0132) (Fig. 2B), response to stimulus (GO: 0050896; p value: 0.3244) *etc*. List of enriched biological processes and their sub-categories in Ladakhi cows is mentioned in Supplementary Table S1. Under GO term ‘response to stimulus’ the major subcategories included were response to stress (GO: 00006950; p value: 0.5850) and immune response (GO: 0006955; p value: 0.8423). Under term ‘response to stress’ (Fig. 2C) several other terms like cellular response to stress; defense response; response to wounding, oxidative stress, starvation and hypoxia were found to be enriched. The genes represented under each subcategory of response to stress in Ladakhi cows are listed in Supplementary Table S2. The top molecular functions identified in Ladakhi cows are mentioned in Supplementary Table S3 included binding (GO: 0005488; p value: 0.5784), transporter activity (GO: 0005215; p value: 0.0933), catalytic activity (GO: 0003824; p value: 0.7139), molecular transducer activity (GO: 0060089; p value: 0.1923), molecular function regulator (GO: 0098772; p value: 0.711) and enzyme regulator activity (GO: 0030234; p value: 0.0330). Enriched sub-categories of molecular functions in Ladakhi cows are shown in Fig. 2D and list of genes identified under sub-categories of molecular functions in Ladakhi cows is mentioned in Supplementary Table S4.

In tropically adapted Sahiwal cows, the major GO terms identified under biological processes are shown in Supplementary Fig. S2A and Supplementary Table S1. Under biological functions in Sahiwal cows response to stimulus, (GO: 0050896; p value: 0.0788), cellular process (GO: 0009987; p value: 0.0634), biological regulation (GO: 0065007; p value: 0.0525) were the most common GO terms observed in the data set. Under response to stimulus, the important sub-ontologies identified were: response to stress, biotic stimulus, abiotic stimulus and immune response. Under response to stress (Fig. S2B) two sub-ontologies identified were cellular response to stress and oxidative stress. Under immune response (Fig. S2C), the major sub-ontologies enriched were innate immune response, adaptive immune response and cell activation involved in immune response. List of genes identified under sub-categories of response to stress and immune response in Sahiwal cows is mentioned in Supplementary Tables S5 and S6. Additionally, the major GO terms identified are shown in Supplementary Fig. S2D and Supplementary Table S3. Several molecular functions like catalytic activity (GO: 0005215; p value: 0.0716), binding (GO: 0005488; p value: 0.3637), transporter activity (GO: 0005215; p value: 0.9907) and enzyme regulatory activity (GO: 0030234; p value: 0.7861) were enriched in Sahiwal transcriptome data. List of genes identified for sub-category, catalytic activity in Sahiwal cows is mentioned in Supplementary Table S7.

### Molecular pathways

A total of 3910 DEGs were utilized to identify the molecular pathways. The metabolic and signaling pathways most impacted in the study (see Supplementary Table S8) were MAPK signaling pathway (p value: 5.85E-04; 14 genes), Electron transport chain (p value: 0.1029; 12 genes) and signaling pathways were apoptosis (p value: 0.0046; 7 genes), IL2 signaling (p value: 0.0665; 7 genes), IL3 signaling (p value: 0.3920; 4 genes), IL6 signaling (p value: 3417; 3 genes), TLR signaling pathway (p value: 0.0329; 4 genes) Delta Notch signaling pathway (p value: 0.0993; 7 genes), Apoptosis modulation and signaling (p value: 0.1373; 5 genes), Myometrial Relaxation and Contraction (p value: 0.1541; 10 genes), TNF-alpha and NF- kB signaling pathway (p value: 0.4421; 7 genes).

### Validation of gene expression data by qPCR

To validate the microarray results quantitative- real time PCR (qPCR) was performed for some hypoxia related target genes such as *HIF-1α*, *EPAS-1*, *VEGF-A*, *ECE*-*1*, *NOS2*, *GLUT-1*, *HK2*, *TNFα*, *GRα* and heat shock proteins; *HSP27, HSP70* and *HSP90*. For data normalization *GAPDH, RPS9, HMBS* and *RPS15* were identified as the most stably expressed internal control genes (ICGs) in all the 30 PBMC samples. The identified ICGs were subsequently utilized to normalize the qPCR data generated for hypoxia and heat stress related genes in PBMCs of different cattle types. The qPCR performance for each ICG and target genes in terms of slope of six-point standard curve, coefficient of determination of standard curve (R^2^) and efficiency of amplification (E = 10^(−1/slope)^) is mentioned in Supplementary Tables S9 and S10.

**Figure 3:**
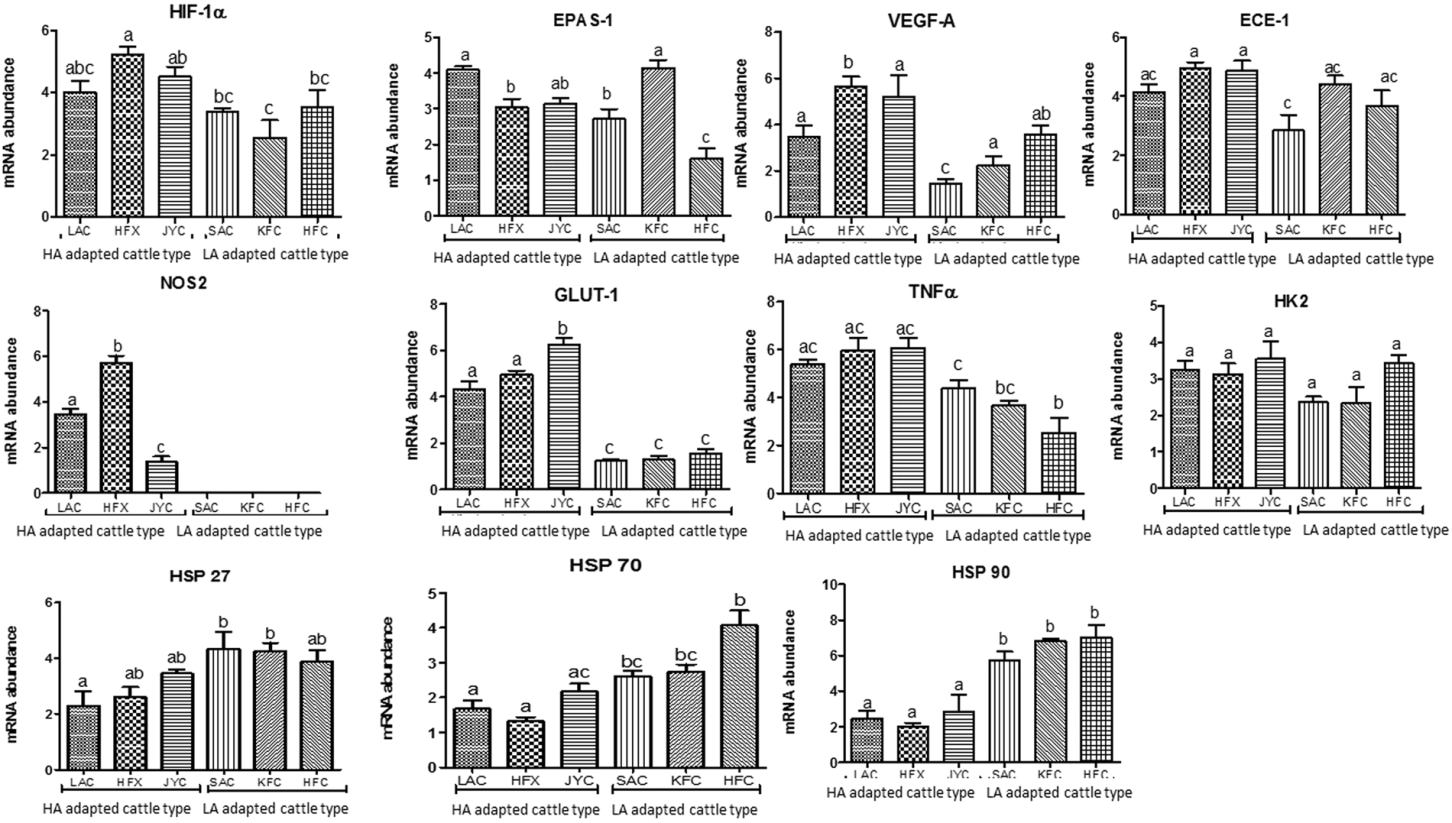
mRNA expression of up-regulated target genes in microarray by qPCR in cattle types adapted to high altitude region and low altitude region. *HIF-1α* (Hypoxia inducible factor-1α), *EPAS-1* (Endothelial PAS Domain 1), *VEGF-A* (Vascular endothelial growth factor-A), *ECE*-*1* (Endothelin converting enzyme 1), *NOS2* (Nitric oxide synthase2), *GLUT1 (*Glucose transporter-1), *HK2* (Hexokinase2), *TNFα* (Tumor necrosis factor α) and *GRα (*Growth receptor *α)*, Heat shock proteins *(HSP27, HSP70* and *HSP 90)*. HA: High altitude, LA: Low altitude. LAC: Ladakhi cattle, HFX: Holstein Frisian cross, JYC: Jersey cattle, SAC: Sahiwal cattle, KFC: Karan fries cattle, HFC: Holstein Frisian cattle.

The qPCR results showed an increased expression of hypoxia related target genes *viz*; *HIF-1α*, *EPAS-1*, *VEGF-A*, *ECE*-*1*, *NOS2*, *GLUT-1*, *HK2*, *TNFα* and *GRα* in cattle types from high altitude region (LAC, HFX, JYC) than cattle types from low altitude (SAC, HFC, KFC). Among high altitude cattle types, Ladakhi cattle showed least expression of all the target genes in comparison to Holstein Friesian crosses and Jersey cattle residing at high altitude (Fig. 3). In comparison to high altitude cattle, the qPCR data showed a significant (p < 0.05) increase in mRNA expression of *HSP27*, *HSP70* and *HSP90* in cattle from low altitude than cattle from high altitude region.

## Discussion

Ladakhi cattle population is hypoxia tolerant, living in an extremely inhospitable high-altitude environment with low temperature, compared to Sahiwal cattle living in extreme hot tropical environment. Out of 3910 DEGs, the number of down-regulated DEGs was quite high in PBMCs of Ladakhi cows (2703) in comparison to Sahiwal cows (1207). This could probably be explained by the fact that high altitude hypoxia environment has a down-regulatory effect on overall transcriptional machinery of Ladakhi cows living at high altitude in comparison to Sahiwal cows living at tropical/normoxic conditions. Variation in the number of DEGs in Ladakhi and Sahiwal cows clearly show the difference in transcriptome profile of both the cattle from two different altitudes. Further, hierarchical clustering, *k*-means clustering and distinct heat maps also revealed that the transcriptome signature of Ladakhi cows is highly distinct from that of the Sahiwal cows.

The genes that were up-regulated in Ladakhi cattle might be responsible for high altitude adaptation. Amongst these, Inhibin beta c (*INHBC*) that was the top most up-regulated gene (197.63 Fold) in Ladakhi cows is involved in regulating cell growth, proliferation, differentiation^11^ and a number of diverse functions such as hypothalamic and pituitary hormone secretion, gonadal hormone secretion, germ cell development and maturation, erythroid differentiation, insulin secretion, nerve cell survival and bone growth. Inositol 1, 4, 5-triphosphate receptor type 1 (*ITPR1*), the second most up-regulated (75.37 Fold) gene in Ladakhi cows is a target of *HIF-2α*^12^ and plays an important role in ER stress-induced apoptosis. Some of the important hypoxia related genes such as *HIF-1 HIF-3α*, *VEGFA*, *VEGFB, VEGFC*, *NOS2*, *GLUT-1*, *GLUT-3* and *HK2* identified in present data set have been marked as important candidate genes in previous studies. Hypoxia inducing factor-alpha (*HIF-1α*), up-regulated 15.66 fold in Ladakhi PBMCs act as a key regulator of O_2_ homeostasis at the cellular and systemic level^13^. The activation of *HIF-1α* is mainly dependent on the hypoxia induced stabilization of the α-subunit, whereas its β-subunit can act independent of the concentration of O_2_. Under hypoxia, activation of the anaerobic glycolysis mainly occurs to compensate for the energy deficit in the cell. *HIF-1α* is known to up-regulate the expression of enzymes involved in glucose uptake (glucose transporters) and glycolysis^14^. In parallel, HIF-1-dependent factors modify the vascular tonus resulting in an improved blood circulation. Till now, about 100 target genes have been shown to be regulated by this gene^15^. Our data showing significant induction of *HIF-1α* gene strongly support the role of *HIF* gene in maintaining the O_2_ homeostasis and other processes necessary for survival of high altitude adapted Ladakhi cows. Simultaneously, hypoxia inducing factor 3A (*HIF3A*) that acts as a transcriptional regulator in adaptive response to low O_2_ tension was also up-regulated 18.53 fold in PBMCs of Ladakhi cow. *HIF* is also known to activate vascular endothelial growth factor (*VEGF*), which encodes for protein that induces angiogenesis^16^. *VEGFs* are essential for growth of new blood vessels promoting angiogenesis and play an important role in hypoxia response and high altitude adaptation^17^. All the three vascular endothelial growth factors, *VEGFA*, *VEFGB* and *VEFGC* were up-regulated in Ladakhi PBMCs by 6.35, 4.75 and 6.93 fold, respectively. Up-regulated expression of *VEGFs* in Ladakhi cows suggest them to be candidate genes in high altitude adaptation. Similarly, Endothelial PAS domain-1 (*EPAS-1*), another major transcription factor was up-regulated (19.72 fold) in Ladakhi cows. The association of *EPAS1* gene in high altitude adaptation is well documented in humans and dogs^18–20^ and has been related to the haemoglobin concentration in Andeans and Tibetans^21–23^. The nitric oxide synthase (*NOS2*) was also up-regulated (17.46 fold) in Ladakhi PBMCs, further substantiating the previous studies that high altitude hypoxia increases the expression of inducible nitric oxide synthase^24^. The nitric oxide (NO) generated from *NOS* is associated with several physiological processes at higher altitude. In the presence of NO, blood vessels inner lining (endothelium) signal the surrounding smooth muscle to relax which results in vasodilation and increase in blood flow to enhance the O_2_ supply under hypoxic condition. The enhanced expression of *NOS2* gene may be responsible for higher production of NO in Ladakhi cattle so as to help in its pulmonary vasculature vasodilation under hypobaric hypoxia condition aiding in better survivability. Calcium channel, voltage-dependent, T type, alpha 1G subunit (*CACNA1G*) that encodes one of the voltage-sensitive calcium channels mediating the entry of calcium into cell was upregulated by 26.51 fold in Ladakhi cattle. This gene is an integral part of calcium signaling pathway and reported to play an important role in high altitude adaptations to hypoxia^25^.

Forkhead box M1 (*FOXM1*) and regulator of telomere elongation helicase (*RTEL1*) genes upregulated in Ladakhi cows have been reported to play an essential role in repairing oxidative DNA damage^26,27^. This gene was also marked as an important candidate gene in Snub nosed monkeys^28^ and Tibetan pigs^29^.

Further, up-regulation of Myosin heavy chain 2 (*MYH2*) gene in Ladakhi cows may be related to skeletal muscle contraction and regulation of processes like glycolysis and glucose metabolism. This gene has also been reported to have higher expression in yak in comparison to cattle of low altitude^30^. Similarly, higher expression of hexokinase 2 (*HK2*) in Ladakhi cows suggested increased glucose metabolism in hypoxia environment as *HK2* is known to be an important glycolytic enzyme involved in the metabolism of glucose to glucose-6-phosphate^31^. Several studies have reported a close association of *HK2* expression with glucose transport^32,33^. Other genes involved in anaerobic metabolism like glucose transporters (*GLUT1*; *GLUT3*), lactate dehydrogenase A (*LDHA*), pyruvate dehydrogenase kinase 1 (*PDK*) were also present in higher abundance in Ladakhi cows. Several hypoxia-responsive genes namely, *MMP1*, *MUC1*, *APLN*, *FZD3* were also upregulated in Ladakhi cows. Amongst these, *APLN* and *FZD3* encode for factors with proangiogenic functions as reported under hypoxic conditions^34^. *PRKAA1*, another important upregulated gene in Ladakhi cows has been reported to be important in achieving genetic adaptation and maintaining metabolic homeostasis at high altitude^35^. Furthermore, the transcript for *SENP2* an enzyme implicated in erythropoiesis^36^; *MICU1*, *VAV3*, *ARNT2* linked to hemoglobin concentration; *CXCL17* involved in vascular physiology and *PAFAH1B3* linked to hypoxia^37,38^ were also observed to be upregulated.

Interestingly, most of the genes from immune functional classes were down-regulated in Ladakhi cows PBMCs. The lower expression of these genes in Ladakhi cows could be explained by the fact that at high altitude environment, there is lesser load of pathogens/microbes. This scenario may reduce the risk of opportunistic infection in Ladakhi animals, hence these immune genes were relatively lowly expressed in comparison to Sahiwal cows that belongs to tropical conditions.

In comparison to high altitude adapted Ladakhi cows, genes like *eEF1A1* responsible in stabilizing the formation of functional ribosome and in translation initiation; chemokine genes such as *CXCL3*, C*CL4*, *CCL16* related to several immune functions and host defence were present several fold higher in Sahiwal PBMCs. The chemokines are known to induce strong immune response in cattle breeds and play fundamental role in the homeostasis, and functioning of immune system^39^. Another major immune related gene *CSF3R*, a member of cytokine receptors family that helps in cell surface adhesion or in migration of leukocytes was observed to be up regulated in Sahiwal PBMCs. The protein encoded by this gene is the receptor for colony stimulating factor 3, a cytokine that controls the production, differentiation, and function of granulocytes. The series of beta defensin genes *viz*., *DEFB3*, *DEFB1*, *DEFB4A* and *DEFB10* involved in host defence system was observed to be upregulated in Sahiwal PBMCs. These antimicrobial peptides are thought to be evolved under the pressure of natural selection to maintain a host-pathogen balance in cattle breeds adapted to tropical region^40^. The higher abundance of these antimicrobial peptides can possibly be explained with the fact that cattle types from tropical region are exposed to diversity of pathogens. Additionally, the enrichment of genes like *BOLA-DQA3, BoLA*, *BOLA DQA1*, *BOLA DQB* and *BOLA DRB3* in PBMCs of Sahiwal cows in comparison of Ladakhi cows signifies the immune potential and active host defence mechanism in Sahiwal cattle to combat the pathogens normally present in the tropical conditions. Overall, these genes would enable the immunological defences of Sahiwal cows under harsh tropical environmental condition. The differences in expression of *BOLA* genes in Sahiwal and Ladakhi cows might be due to the differences in immune system activation at two distinct altitudes and types of pathogens prevailing in high and low altitude environment. In addition, increased expression of molecular chaperons such as *DNAJC2*, *DNAJC27*, *HSPA4*, *HSPB11* in Sahiwal than Ladakhi, could be due to environmental heat load present in the tropical conditions in comparison to environmental condition (cold arid) prevalent in Leh and Ladakh.

The overexpression of cluster of genes related to DEAD Box polypeptides (*DDX*), *DDX47*, *DDX55, DDX6*, *DDX18*, *DDX21*, *DDX23*, *DDX54*, and *DDX59* was also significant in Sahiwal than Ladakhi cows. These genes are known to be highly conserved in nature and are generally involved in RNA metabolism. The results clearly suggest that there could be substantial differences in the processes or expression of proteins involved in RNA metabolism between tropically adapted Sahiwal and high altitude adapted Ladakhi cows. The lower abundance of DDX genes in Ladakhi cows from high altitude is similar to few other studies that have shown that under hypoxic condition, there is down-regulation of DDX expression^41^. Also significant increase in expression of several ribosomal proteins *viz*; *RPL34, RPL36*, *RPS12*, *RPS24*, and mitochondrial ribosomal proteins *MRPS15 MRPL44, MRPL46, MRPS17* could be due to enhanced need of ribosomal biogenesis to maintain normal protein translation and essential cellular functions in Sahiwal cows. Overall, the transcriptome data generated for Sahiwal cows PBMCs indicated the comparative enrichment of genes associated with immune function, inflammatory response, heat stress, biological process regulation and ribosomal biogenesis related genes.

Gene ontology analysis revealed that genes involved in biological process like cellular response to stimulus (GO: 0051716; p value: 0.133; 74 genes), regulation of biological process (GO: 0050789; p value: 0.228; 152 genes), regulation of biological quality (GO:0065008; p value: 0.115; 43 genes), response to stress (GO:0006950; p value: 0.585; 40 genes) and response to hypoxia (GO: 0001666; p value: 0.660; 11 genes) are likely to play a role in the high altitude adaptation of Ladakhi cattle. Additionally, some of the interesting biological terms *viz*., homeostasis process (GO :0042592; p value: 0.164; 34 genes), regulation of body fluid levels (GO: 0050878; p value: 0.003; 7 genes), regulation of anatomical structure (GO:0090066; p value: 0.348; 8 genes), regulation of blood pressure (GO:0008217; p value: 0.702; 3 genes), regulation of hormone level (GO:0010817; p value: 0.831; 7 genes), regulation of protein stability (GO:0031647; p value: 0.885; 3 genes) were found to be enriched in Ladakhi cows. Similarly, a large number of molecular functions like binding (GO: 0005488; p value: 0.578; 187 genes), oxidoreductase activity (GO: 0016491; p value: 0.431; 22 genes), transmembrane transporter activity (GO: 0022857; p value: 0.034; 23 genes), signal transducer activity (GO: 0004871; p value: 0.295; 18 genes), enzyme regulator activity (GO: 0030234; p value: 0.032; 21 genes) were also found to be enriched in Ladakhi cows. Overall, the genes identified in the present data set along with enriched biological and molecular functions provide genetic and physiological adaptation to Ladakhi cattle and may be considered as signature of high altitude/hypoxia adaptation.

The Sahiwal cattle though well adapted to tropical conditions, its comparison with Ladakhi cattle from colder climate of Ladakh region revealed higher abundance of genes related to response to stimulus, (GO: 0050896; p value: 0.078; 202 genes) and immune system process (GO: 0002376; p value: 0.070; 53 genes). Under response to stimulus, genes were related to response to stress (GO: 0006950; p value: 3.78E-04; 113 genes), response to abiotic stimulus (GO: 0009628; p value: 0.084; 29 genes) and immune response (GO: 0006955; p value: 0.025; 34 genes). Under immune response, upregulated genes were mainly involved in innate immune response (GO: 0045087; p value: 0.106; 18 genes), adaptive immune response (GO: 0002250; p value: 0.221; 7 genes) and cell activation involved in immune response (GO: 0002263; p value: 0.368; 5 genes). The enrichment of these GO terms in high environmental temperature and humidity conditions prevalent in the sampling region of Sahiwal cows was expected. This could be attributed to the typical arid or semiarid environmental condition exist in the region of sampling for Sahiwal cows and constantly exposed to tropical pathogens.

**Figure 4:**
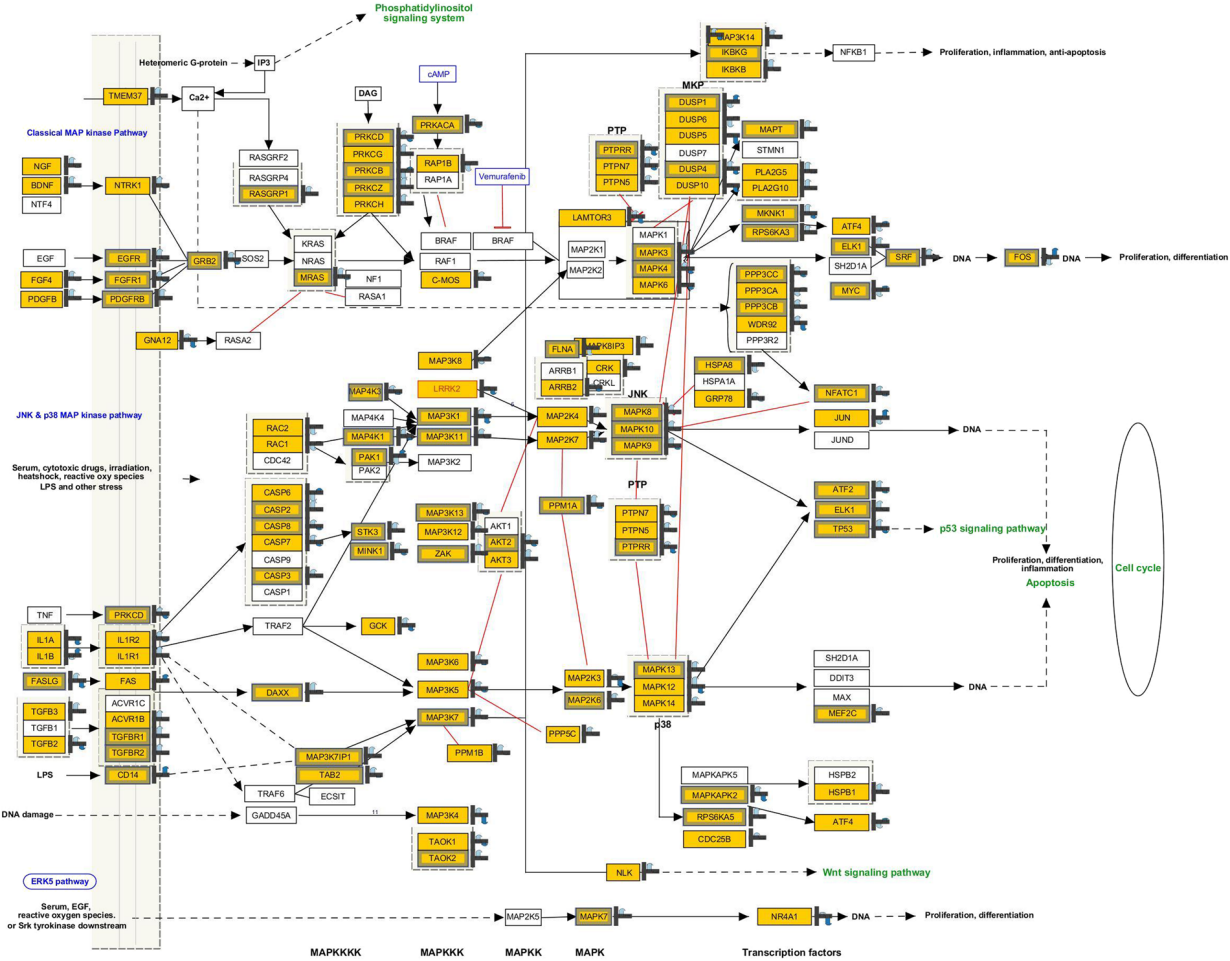
MAPK Signaling pathway showing expression pattern of DEGs in Ladakhi and Sahiwal cows. This particular pathway is known to be involved in triggering the cellular response to environmental stress. A total of 39 genes were matched in the pathway entity list. Genes in yellow color represent matched entities.

The molecular pathways impacted in both the cattle types adapted to extreme agro-climatic regions included MAPK signaling pathway, Electron transport chain, apoptosis, IL2 signaling, IL3 signaling, IL6 signaling, TLR signaling pathway, Delta Notch signaling pathway, Myometrial relaxation and contraction and TNF-alpha and NF- kB signaling pathway. Among these MAPK signaling pathway known to be involved in triggering the cellular response to environmental stress was the most impacted pathway. A total of 39 genes (14 up and 25 down regulated) were matched in the pathway entity list (Fig. 4). The upregulated genes *TAB2, TGFB2, MAP4K1, MINK1, ATF2, MAPT, ARRB2, MAPK3, KRAS, CDC25B, PTPN7, JUND, MAP4K3* of this particular pathway in Ladakhi cows may be associated with the high-altitude adaptation. Some of the recent studies have also shown indication of MAPK signaling pathway in high altitude adaptation especially in Tibetans^42^. Further, in recent past, relationship of this pathway with hypoxic response has also been documented^43^. Taken together, the series of pathways impacted in the present data set along with distinct transcriptome profile under hypoxic (high altitude) and normoxic (mean sea level) conditions indicated signatures of adaptive evolution of these two cattle types in response to diverse environments.

The qPCR analysis also substantiated higher expression of hypoxia related target genes such as *HIF-1α, EPAS-1, VEGFA, NOS2, ECE-1, GLUT1, HK2*, *TNFα, GRα* in PBMCs of Ladakhi cattle in comparison to breed from low altitude region suggesting pivotal role of these genes in high altitude adaptation. The enrichment of genes reported for high altitude adaptation in few other species like, Tibetan antelopes, Tibetan wild boars, yaks and ground tits seems to be corroborated with the present findings, indicating existence of common evolutionary mechanism for high altitude adaptation^44–47^. Based on the generated data, local native Ladakhi cattle are better adapted to withstand the hypoxic condition than exotic or cross-bred populations. The data indicated that hypoxia related genes get accumulated under hypoxic conditions and probably are essential adaptive component for the animals surviving at high altitude. On the other hand, significantly higher expression of molecular chaperons *HSP27*, *HSP70* and *HSP90* genes in tropically adapted Sahiwal cattle than Ladakhi and other exotic breeds from high altitude could be due to induction of molecular chaperons at high ambient temperature prevalent under tropical habitat. Overall, the expression pattern of several target genes generated by qPCR corroborated well with the microarray data set.

In conclusion, the comparative data analysis indicated distinct transcriptome signature of two cattle types native to extreme climatic conditions. Based on data generated in the present study, native cattle of Ladakh region was found to be genetically distinct from native cattle adapted to tropical region of India

## Methods

### Ethics statement and animal selection

The blood sampling of animals were performed in accordance with the relevant guidelines and regulations as approved by Institutional Animal Ethics Committee (IAEC) of National Bureau of Animal Genetics Resources (NBAGR), Karnal. In the present study, transcriptome profile was generated in a total of 11 PBMCs samples; 5 heifers of Ladakhi cattle adapted to high altitude hypoxic condition; and 6 heifers of Sahiwal cattle from low altitude normoxic condition. The sampling of both the cattle types was accomplished in the month of August. About 25–30 ml of whole blood was collected aseptically from external jugular vein in sterile EDTA coated vacutainer tubes. The blood samples were then taken to laboratory in an icebox and were subjected for PBMC isolation.

### PBMC isolation and RNA extraction

The PBMCs were isolated from the whole blood using density gradient centrifugation method as described in our previous reports^48^. In brief, whole blood was diluted with 1 × PBS (Ca^2+^ and Mg^2+^ free; Hyclone, Utah) in 1:1 ratio and gently over laid on Histopaque-1077 (Sigma-Aldrich Inc., USA) and centrifuged at 400 RPM for 30 mins at room temperature. After removing the buffy coat, lymphocyte pellet was treated with 2 ml of chilled RBC lysis buffer. The reaction was stopped by adding 8.0 ml of 1 × PBS (Ca^2+^ and Mg^2+^ free; Hyclone, Utah) followed by centrifugation at 260 g for 10 min. After getting a clear white pellet, the cells were washed twice with 1 × PBS and were suspended in 1.0 ml of ice cold Trizol reagent (Invitrogen, Carlsbad, California). Total RNA was extracted and purified from 11 PBMC samples using ice cold Trizol reagent according to the manufacturer’s instructions (Invitrogen, Corp., CA). The traces of genomic DNA were removed by RNase free DNase treatment (Qiagen, Germany) using RNeasy Mini kit columns (Qiagen, Germany). The quality and concentration of extracted RNA was measured using NanoDrop ND-1000 spectrophotometer (NanoDrop Technologies) and bioanalyzer (Bio-Rad, USA).

### cRNA labeling and hybridization with bovine 44 K oligonucleotide chip

**Figure 5:**
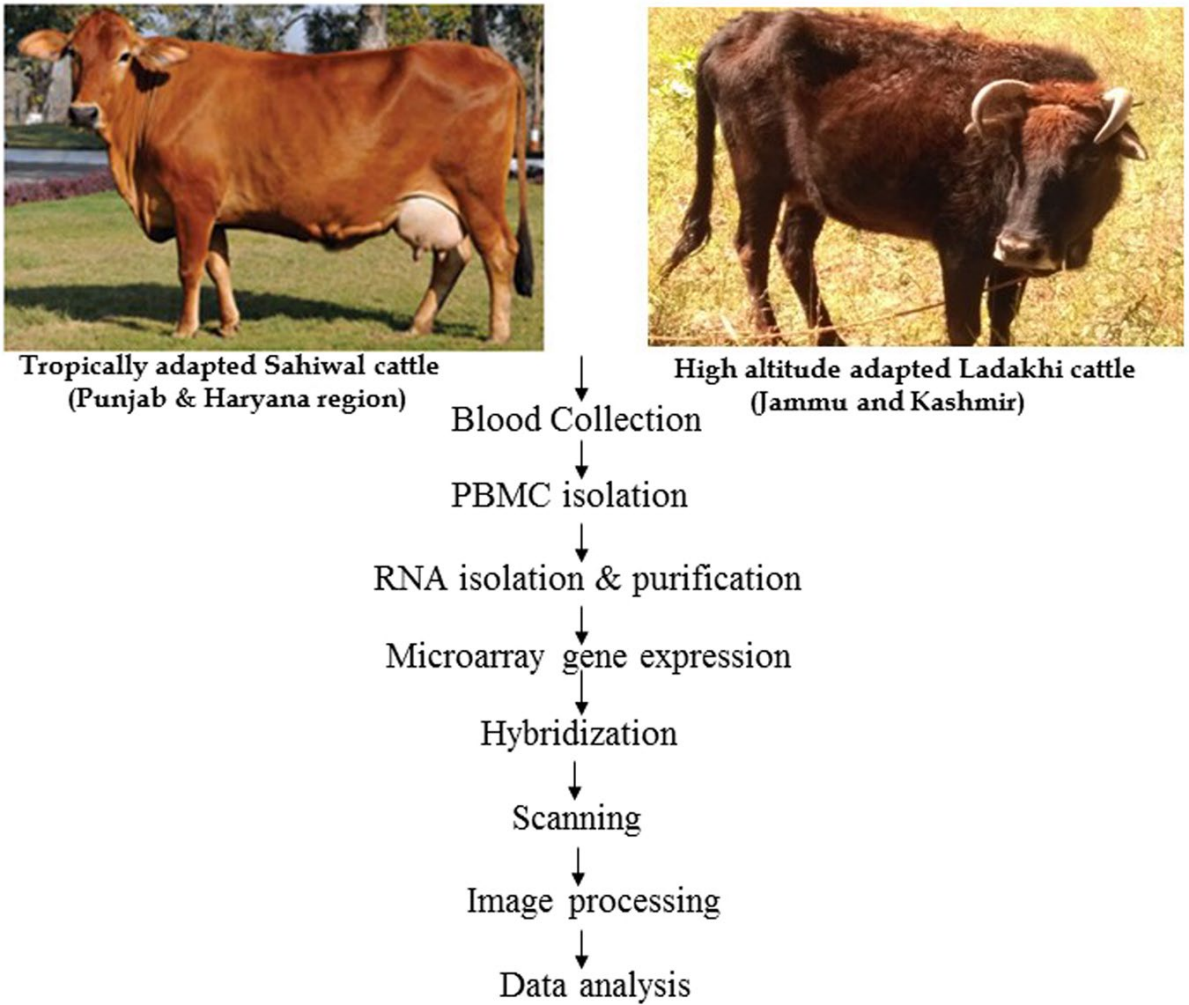
Workflow of microarray experiment and data analysis.

The transcriptome signature was generated using Agilent whole genome bovine 44 K chip harbouring 60 mer oligos and 21520 entities. The entire microarray related procedures *viz*; labeling, hybridization, washing and scanning were followed as described in our previous study^49^. The entire procedure is shown in Fig. 5. Labeling and microarray processing was performed as per “One color protocol using Agilent’s low input quick amp labeling kit” (Agilent Technologies, Santa Clara, CA). 2 µl of total RNA (100 ng/µl) samples were labeled with T7 promoter primer at 65 °C for 10 min and incubated in ice for 5 min. cDNA was constructed from labeled RNA samples after adding cDNA master mix (5× First Strand Buffer, 0.1 M DTT, 10 mM dNTP mix and Affinity Script RNase Block Mix) followed by incubation at 40 °C for 2 h, 70 °C for 15 min and final incubation on ice for 5 min. The cDNA was reverse transcribed to synthesize cRNA (complimentary RNA) and was amplified by adding transcription master mix (5× Transcription Buffer, 0.1 M DTT, NTP mix, T7 RNA Polymerase Blend and Cyanine 3-CTP) followed by incubation at 40 °C for 2 h. The amplified labeled cRNA was purified (RNeasy mini column kit, Qiagen, Germany), and quantified to obtain the yield of cRNA {μg cRNA yield = (Concentration of cRNA) ×30 μl (elution volume)/1000} and specific activity of Cy3 {pmol Cy3 per μg of cRNA = (Concentration of Cy3/Concentration of cRNA) ×1000}. For hybridization, 1.65 μg of linearly amplified and Cy3-labeled cRNA were fragmented using fragmentation mix (10× Blocking Agent and 25× Fragmentation Buffer) and incubated at 60 °C for exactly 30 min. After incubation the fragmented RNA samples were immediately transferred on ice for one min and added 2× GEx hybridization buffer to stop the reaction. The fragmented samples were carefully dispensed to the array on gasket slide placed in hybridization chamber base without introducing air bubble. Slowly placed an array onto the gasket slide with “Agilent labeled barcode facing down and the numeric barcode facing up. Placed the assembled slide chamber in rotisserie in a hybridization oven allowing it to rotate at 10 rpm for 17 hrs at 65 °C. Post hybridization, the microarray slides were disassembled in gene expression (GE) wash buffer 1 (pre warmed overnight at 37 °C) containing 0.005% Triton-X to reduce the possibility of array wash artifacts. This was followed by second wash with GE Wash Buffer 2 for 5 min at room temperature.

### Scanning, data acquisition and analysis

The slides were scanned immediately after washing on Agilent DNA Microarray Scanner (G2505B) using one colour scan setting. The signal intensities were checked from the digit images using Agilent Feature Extractor Software Version 9.5 (Agilent Technologies). The microarray raw data files in.txt format were imported to GeneSpring GX 13.1 (Agilent Technologies) and subjected for quantile normalization which is highly effective in reducing variation between arrays. Normalized data was analysed for statistically significant gene expression differences between Ladakhi cows and Sahiwal cows to the fold change statistic that uses overall gene expression variation to calculate a gene-specific variance. To keep the number of false positives to alpha = 0.05, a false discovery rate adjustment was used^50^. Genes that were statistically significant in expression between Ladakhi cows and Sahiwal cows were grouped according to fold expression (up- vs. down-regulation). The data was further subjected to hierarchical clustering and *k-*means clustering based on entities (genes), gene ontology enrichment analysis and pathway analysis.

### Microarray expression data validation by qPCR

The microarray data was further validated by comparing the expression of some of the up-regulated hypoxia related genes such as *HIF-1α*, *EPAS-1*, *VEGF-A*, *ECE*-*1*, *NOS2*, *GLUT-1*, *HK2* and heat shock proteins (*HSP27, HSP70 & HSP90*) in cattle types from high altitude region and tropical region. For this a total of 30 PBMC samples were selected; 5 each of Ladakhi cows (LAC), Holstein Frisian crosses (HFX), Jersey cows (JYC) from high altitude region; and Sahiwal cows (SAC), Karan Fries cows (KFC), Holstein Friesian cows (HFC) from tropical region using qPCR. For optimal gene expression analysis, qPCR data of target genes was normalized using a total of 10 previously known candidate reference genes from different functional categories *viz*., *GAPDH*, *RPL4*, *EEF1A1*, *RPS9*, *HPRT*, *UXT*, *HMBS*, *B2M*, *RPS15* and *ACTB*. The primers for each of the ICGs and target genes were either selected from literature or designed using Primer express 3.0 software (Applied Biosystem). Primer details for all the ICGs and target genes are provided as supplementary information (Tables S7 and S8). The accuracy of primer pairs was also ensured by the presence of a unique peak during the dissociation step at the end of qPCR cycle. The qPCR was performed using Light Cycler 480 instrument (Roche, Germany) as described in our previous reports^51^. The data was acquired using the ‘second derivative maximum’ method as computed by the Light Cycler Software 3.5 (Roche Diagnostics) and subjected for subsequent analysis.

### Data normalization

qPCR data of target genes in high altitude and tropically adapted cattle was normalized by selecting best suited ICGs utilizing geNorm, NormFinder and Best keeper softwares^52–54^.The mean value was calculated using relative quantification 2^−ΔΔCT^ method^55^. The differences between groups were tested by Tukey’s Multiple Comparison Test. P values less than 0.05 and 0.01 were considered significant. Statistical test was performed using GraphPad PRISM version 5.0 (La Jolla, CA, USA).

## Electronic supplementary material

Supplementary Information
