# Supplementary material for: Transcriptome Analysis of Circulating PBMCs to Understand Mechanism of High Altitude Adaptation in Native Cattle of Ladakh Region
Source: Sci Rep. 2018 May 16;8:7681. doi: 10.1038/s41598-018-25736-7 (PMC5955995; doi:10.1038/s41598-018-25736-7)
Supplement: Supplementary file 1 — Supplementary Information [file 41598_2018_25736_MOESM1_ESM.pdf]

Transcriptome Analysis of Circulating PBMCs to Understand Mechanism of High Altitude Adaptation in Native Cattle of Ladakh Region

Preeti Verma[1^], Ankita Sharma[1], Monika Sodhi[1], Kiran Thakur[1], RS Kataria[1], SK Niranjan[1], Vijay K Bharti[#], Prabhat Kumar[#], Arup Giri[#], Sahil Kalia[#] and Manishi Mukesh[*1]

Supplementary Figures

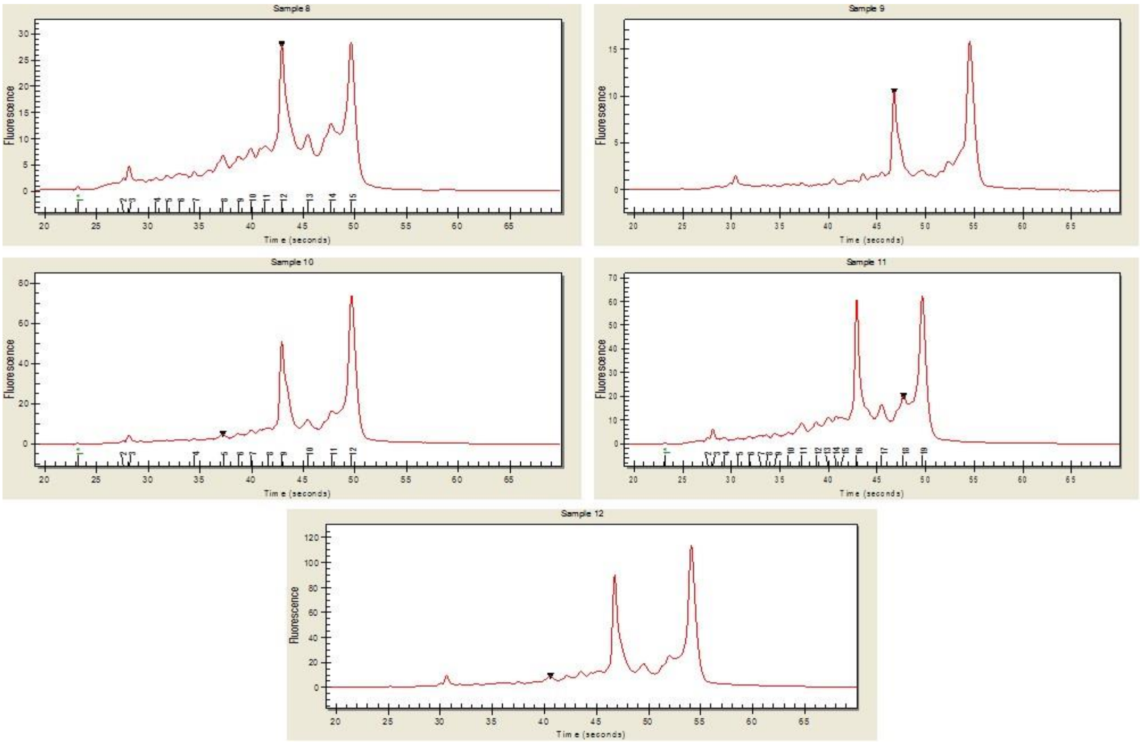

Figure S1: The virtual image of RNA samples obtained from Bioanalyzer. The electropherograms for each RNA samples showed two characteristics peaks of 28S rRNA &18S rRNA indicating the intactness of the extracted RNA.

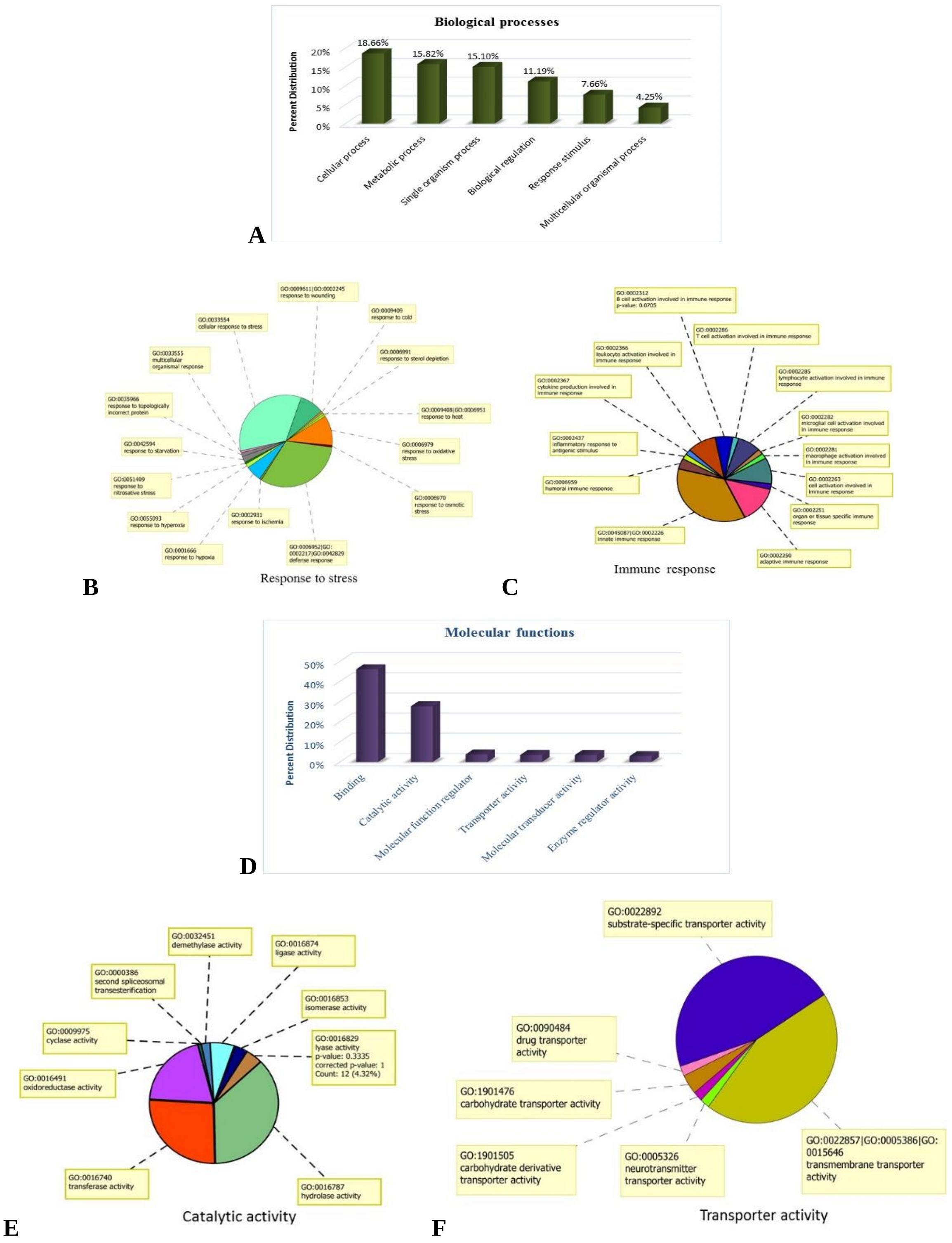

Figure S2: GO analysis of up-regulated genes in Sahiwal cows (A) Enriched biological processes in Sahiwal cows; **(B)** Enriched biological processes under sub category “Response to stress” in Sahiwal cows; **(C)** Enriched biological processes under sub category “immune response” in Sahiwal cows; **(D)** Enriched molecular functions in Sahiwal cows; **(E)** Enriched molecular functions under category “catalytic activity” in Sahiwal cows; **(F)** Enriched molecular functions under category “transporter activity” in Sahiwal cows.

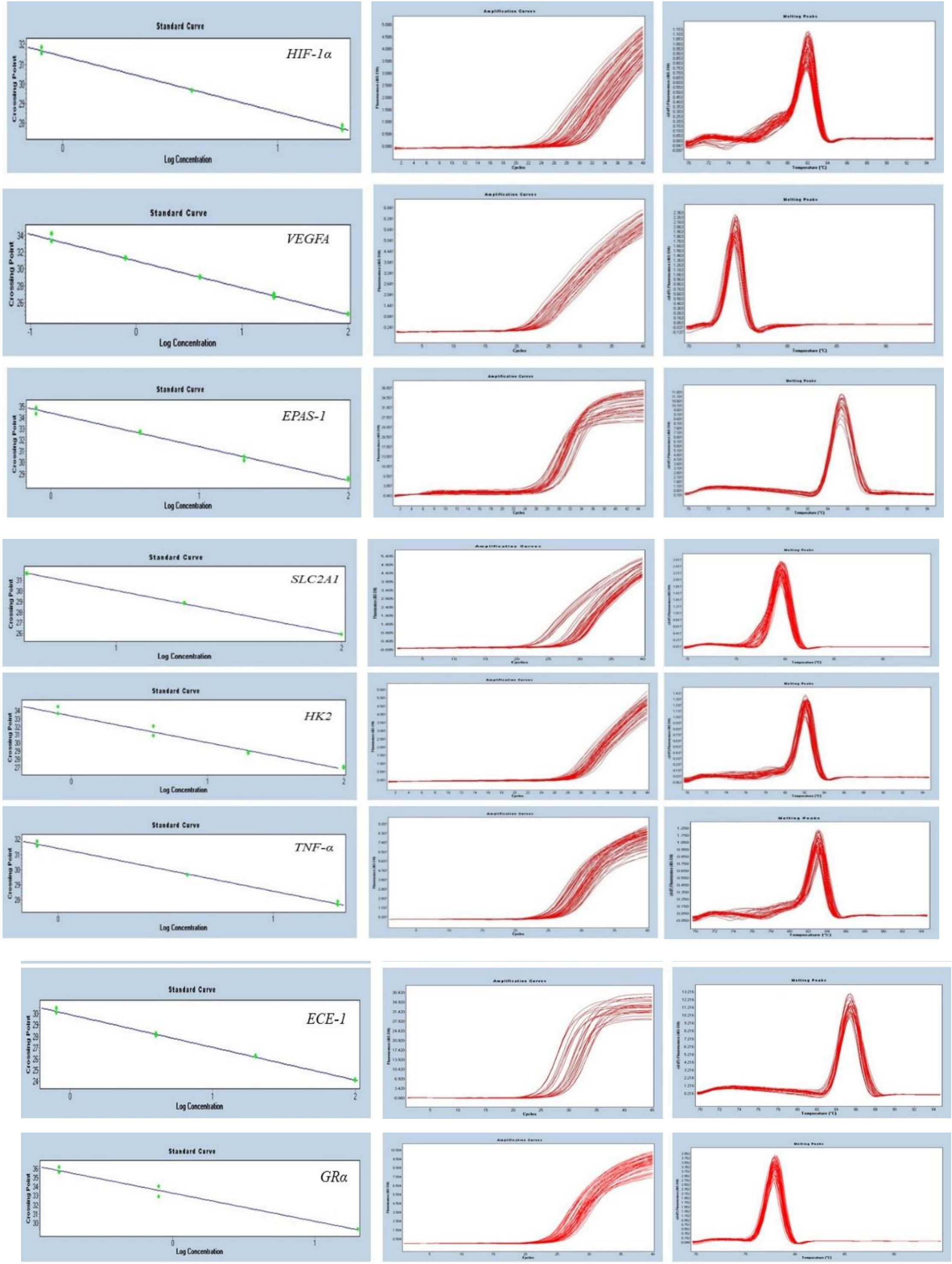
Standard Curve
Amplification Curves
Melting Peaks
HIF-1α
VEGFA
EPAS-1
SLC2A1
HK2
TNF-α
ECE-1
GRα
Crossing Point
Log Concentration

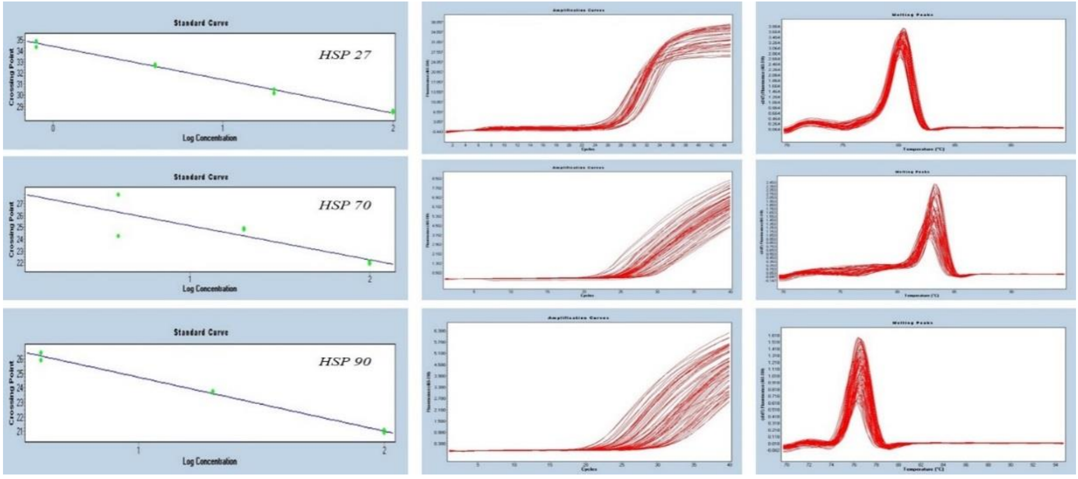

Figure S3: qPCR standard curve, amplification plot and melting peak for high altitude target genes. *HIF-1α* (Hypoxia inducible factor-1α), *EPAS-1* (Endothelial PAS Domain 1), *VEGF-A* (Vascular endothelial growth factor-A), *ECE-1* (Endothelin converting enzyme 1), *SLC2A1 (*Glucose transporter-1), *HK2* (Hexokinase2), *TNFα* (Tumor necrosis factor α) and *GRα* (Growth receptor *α),* Heat shock proteins *(HSP27, HSP70* and *HSP 90).*

Supplementary Tables

Table S1. List of enriched biological processes in high altitude adapted Ladakhi cows and tropically adapted Sahiwal cows

**Ladakhi Cows**		
**Biological process**	**GO ID**	**Gene count**
**Cellular process**	GO:0009987	236
Cellular metabolic process	GO:0044237	129
Single organism cellular process	GO:0044699	215
Cellular response to stimulus	GO:0050896	74
**Response to stimulus**	GO:0050896	94
Cellular response to stimulus	GO:0044237	74
Response to chemical	GO:0042221	34
Response to endogenous stimulus	GO:0009719	10
Response to biotic stimulus	GO:0009607	4
Immune response	GO:0006955	9
Response to stress	GO:0006950	40
Response to external stimulus	GO:0009605	12
**Signaling**	GO:0023052	53
Single organism signaling	GO:0044700	53
Synaptic transmission	GO:0007268	1
**Metabolic process**	GO:0008152	175
Primary metabolic process	GO:0044238	136
Cellular metabolic process	GO:0044237	129
Catalytic activity	GO:0003824	110
Nitrogen compound metabolic process	GO:0006807	76
Biosynthetic process	GO:0009058	168
**Biological regulation**	GO:0065007	163
Regulation of biological quality	GO:0065008	43
Regulation of molecular function	GO:0065009	42
Regulation of biological process	GO:0050789	152
**Single-organism process**	GO:0044699	215
Single organism cellular process	GO:0044763	186
Single organism signaling	GO:0044700	53
Single organism transport	GO:0044765	48

Single organism metabolic process	GO:0044710	84
**Sahiwal cows**		
**Response to stimulus**	GO:0050896	202
Cellular response to stimulus	GO:0051716	145
Cellular response to Chemical	GO:0042221	74
Response to stress	GO:0006950	113
Immune response	GO:0006955	34
Response to biotic stimulus	GO:0009607	26
Response to abiotic stimulus	GO:0009628	29
**Single organism process**	GO:0044699	398
Single organism cellular process	GO:0044763	337
Single organism metabolic process	GO:0044710	185
Single multicellular organism process	GO:0044707	109
Single organism development process	GO:0044767	108
**Multicellular organismal process**	GO:0032501	112
Single multicellular organism process	GO:0044707	109
Multi- Multicellular organism process	GO:0032504	14
Multicellular organism reproduction	GO:0044706	3
**Cellular process**	GO:0009987	492
Cellular metabolic process	GO:0044237	342
Cellular component organization	GO:0016043	144
Cellular response to stimuli	GO:0051716	145
**Biological regulation**	GO:0065007	**295**
Regulation of biological quality	GO:0065008	71
Regulation of biological process	GO:0050789	276
Regulation of molecular function	GO:0065009	70

Table S2: List of genes enriched under sub-category, response to stress in Ladakhi cows

**Response to stress**	**GO ID**	**Genes**
Cellular response to stress	GO:0033554	*CIB1, BRCC3, TDP2, TONSL, SIRT4, TFPT, PPARGC1A, MMS22L, COL4A3BP, CLPB, SPRTN, ERCC2, SLC39A4, UBA5, PKD2, RAD51, CAV1, ALKBH8, PARPBP, RAD9B, SMC1A, ALDH3B1,SETMAR, IFNG, INIP, CREB3, MAD2L2, YOD1, ALKBH2, ERCC6L2, ITPR1, OXR1, MCM8, SMARCAD1, INHBB, ITPR1, ITPR1, BCL2L2, CAPN3, MIF, BRCC3, RTEL1, TP53, VEGFA, ITPR1, GTF2H5, STX17, DDB1, IKBKG, BRE, RNF8, SMARCAL1, CAV1, AQP2, DDX1, ING4, ERLIN2, ATG12, GAB1, CDIP1, ATG5, STX17, MAD2L2, ERLIN2, CAV1, KRT20, PSME4, MAP3K7, CREB3, TRIP12, OS9, ERO1L, NR4A2, SLC2A1, TDP2, SMARCB1, DDB2,MARF1, ASCC3, RNF168, SMARCAD1, ERO1L, MARF1, ERCC2, TERF2IP, RAD21, FBXO6, ITPR1*
Defense response	GO:0006952	*CD46, TBXA2R, TLR9, CXCL11, LBP, LEAP2, GNG7, SERPINF2, ITIH4, C7, RARRES2, CXCR3, KIT, CD40LG, PTGER3, DEFB1, CXADR, KLKB1, IRAK1, TLR3, LOC781146, CNPY3, SMAD1, IFNG, DEFB119, IFNAC, KNG1, ADORA3, CCL20, C4A, CATHL1, DRD1, IFNAC, LYZ1, MX1, FN1, TAP, DEFB1, TLR10, TLR6, MIF, TLR4, AQP4, TOLLIP, DEFB119, C4A, CD14, LY96, SAA4, SAA2, C2, C9, ATG12, IL34, ATG5, AHSG, C1S, CD46, LBP, CLEC7A, C3, CFH, RIPK2, CNPY3, POLR3A, CEBPG, C5*
Response to oxidative stress	GO:0006979	*PTPRN, PPARGC1A, ERCC2, ALDH3B1, CRYAB, OXR1, PTGS2, GPX3, PRNP, APP, APOE, GPX8, APP, GAB1, PTGS1, NR4A2, ERCC2*
Response to hypoxia	GO:0001666	*KCNMA1, AJUBA, CAV1, CLDN3, CRYAB, USF1, VEGFA, CAV1, NR4A2, LOXL2, USF1*

Response to starvation	GO:0042594	*GCG, SLC39A4, CAV1, INHBB, STX17, CAV1, ATG12, ATG5, STX17, CAV1, SLC2A1*	

Table S3. List of molecular functions enriched in high altitude adapted Ladakhi cows and tropically adapted Sahiwal cows.

**Ladakhi Cows**		
**Molecular functions**	**GO ID**	**Gene count**
**Binding**	GO:0005488	187
Ion binding	GO:0043167	92
Organic cyclic compound	GO:0097159	76
Protein binding	GO:0005515	94
Heterocyclic compound binding	GO:1901363	74
Small molecule binding	GO:0036094	43
Lipid binding	GO:0008289	14
**Catalytic activity**	GO:0003824	110
Hydrolase activity	GO:0016787	50
Transferase activity	GO:0016740	28
Oxidoreductase activity	GO:0016491	22
Ligase activity	GO:0016874	08
**Transporter activity**	GO:0005215	27
Substrate-specific transporter activity	GO:0022892	22
Transmembrane transporter activity	GO:0022857	23
**Molecular Transducer activity**	GO:0060089	22
Signal transducer activity	GO:0004871	18
Receptor activity	GO:0004872	15
**Molecular function regulator**	GO:0098772	23
Enzyme regulator activity	GO:0030234	21
Channel regulator activity	GO:0016247	2
**Enzyme regulator activity**	GO:0030234	21
Enzyme activator activity	GO:0008047	13
Enzyme inhibitor activity	GO:0004857	7
Nucleoside triphosphate regulator binding	GO:0060589	6
Peptidase regulator activity	GO:0061134	4
Kinase regulator activity	GO:0019207	4
**Sahiwal Cows**		
**Molecular function**	**GO ID**	**Gene count**
**Binding**	GO:0005488	374
Protein binding	GO:0005515	145
Heterocyclic compound binding	GO:1901363	203
Enzyme binding	GO:0019899	28
Transcription factor binding	GO:0008134	10
Nucleic acid binding	GO:0003676	112
Ion binding	GO:0043167	227
**Catalytic activity**	GO:0016491	254
Oxidoreductase activity	GO:0016491	56
Hydrolase activity	GO:0016787	102
Transferase activity	GO:0016740	73
**Molecular function regulator**	GO:0098772	32
Enzyme regulator activity	GO:0030234	25
Guanyl nucleotide exchange factor	GO:0005085	5
Channel regulator activity	GO:0016247	2
**Molecular transducer activity**	GO:0060089	30
Signal transducer activity	GO:0004871	22
Receptor activity	GO:0004872	25
**Transporter activity**	GO:0005215	30
Transmembrane transporter activity	GO:0022857	24
Substrate specific transporter activity	GO:0022892	24

Table S4: List of genes identified under sub-categories of molecular functions in high altitude adapted Ladakhi cows

Molecular Function	GO ID	Genes
Hydrolase activity	GO:0016787	*F9, DPP4, GNB2, MMP1, NT5E, ABCC1, IMPA1, MYO10, PCSK2, PDE1A, PLCB4, UCHL5, ENTPD1, MAN2B1, UQCRC2, RAB27B, PAG2, ATP5D, PPAP2C, SMPD1, KIF22, GNL3L, CPB2, DOM3Z, PNPLA2, ACAP1, CTDNEP1, ABHD4, ABHD1, SLX1A, HAGHL, RNASEK, GTPBP1, USP1, CDC25A, LPPR2, PSPH, LHPP, CLPP, CPPED1, PNKD, TMEM55A, PTRHD1, ATP5E, F7, PHPT1, KIF2A, ABHD8, PLCXD3, MYH2*
Transferase activity	GO:0016740	*MET, HAS2, LRAT, PIK3CA, FUT4, UBA1, COL4A3BP, GSTM1 ST3GAL5, ST6GALNAC6, ZDHHC16, PFKM, PFKL, ZNRF1, DPM3, LTC4S, PHKG2, ZDHHC6, METTL17, B4GALT3, RABGGTA, AK4, UBE4A, CDK2, TGFB2, RPN2, SUMO3, PRPS1*
Oxidoreductase activity	GO:0016740	*COX7B, LDHA, CYP11B1, PRDX2, COX7A2, NDUFA4, ND1, ND2, ND4L, ND4, ND5, CYTB, NDUFA7, NDUFS7, GFOD2, LTC4S, PYCRL, ACOX1, SDR42E1, OXR1, PYCR1, IDH3G*
Enzyme regulator activity	GO:0030234	*SERPING1, GHRL, IGF2, AGTR1, TIMP1, UCHL5, PIK3CA, ARPP19, SERPINA14, ARHGDIB, ARHGDIA, CDC42EP2, CAP1, ARHGAP10, LTC4S, CLPS, CSNK2B, PSMD2, RASAL3, PPP1R35, APOC2*
Ion binding	GO:0043167	*F9, MET, MMP1, NT5E, SARS, WARS, ABCC1, BGLAP, CNGB1, IMPA1, MYO10, NRXN1, PDE1A, PLCB4, SPARC, TIMP1, ENTPD1, MAN2B1, PIK3CA, UQCRC2, CYP11B1, SLC34A2, RAB27B, ADIPOQ, UBA1, COL4A3BP, ITPR1 CYTB, NDUFS7, ESR1,RPL37A, MOB3A, NUCB1, SCNM1, ZDHHC16, KIF22, PFKM, DRG2, GNL3L, TCEA2, CPB2, DOM3Z, ACAP1, PFKL, ZNRF1, CIB1, SLX1A, HAGHL TRAFD1, PHKG2, MTA3, GTPBP1, ACOX1, ZDHHC6, ASNS, ZFYVE26, MAP1LC3A, QARS, METTL17, B4GALT3, ABGGTA AK4, TCHHL1, ARL6, CDK2, ARL5A, EIF2S2, CYB561D2, SNX17, PLEKHA2, PSPH, LHPP, FSTL1, CIAPIN1, FBLN5, CPPED1, FAM188A, CSNK2B, DGCR8, IKZF3, CRIP1 IDH3G, NARS, ZMAT5, PNKD, F7, KIF2A, CYTH2, ACSF2, PRPS1, MYH2*
Transmembrane transporter activity	GO:0022857	*COX7B, SV2A, AQP4, ABCC1, CHRNE, GABRA4, GABRB1, SLC6A9, SLC34A2, CLIC4, ITPR1, COX7A2, NDUFA4, ATP5D, SLC39A3, SFXN3, ATP5L, SLC17A7, ATP6V0C, TMEM38B, ATP5E, GPR89, AQP3*

Table S5. List of genes enriched under sub-category, response to stress in Sahiwal cows

Function	GO ID	Genes
Cellular response to stress	GO:0006950	*BAX, FOS, SOD2, PIK3R1, SLC2A1, FAS, PSEN1, VAPB, SMARCAL1, APTX, PPIF, NEIL2, COQ7, FAM175A, UBE2T,FXN, MPV17, TDP2, MCTS1, SCAMP5, ATF4, MYC, PRIMPOL, SETMAR, PDIA5, INSIG1, UBE2B, SLU7, BCCIP, PAXIP1, HMOX1, MGARP, RHNO1, ALKBH3, SGK1, NSMCE4A, PSME4, MSH2, MMS19, NABP2, ALKBH7, RAD23A, UBE2W, MACROD1, DERL3, ERCC1, INIP, ATG12*
Response to wounding	GO:0009611	*F5, BAX, SOD2, THBD, ANXA8L1, PROCR, ADRA2A, HMOX1, AJUBA, F2R, PAK1, F13A1*
Response to oxidative stress	GO:0006979	*FOS, APOE, SOD2, PSEN1, PPIF, COQ7, FXN, MPV17, HMOX1, GLRX2, HYAL1, GPX7, PSMB5, ERCC1, ETFDH*

Defense response	GO:0006952	*DEFB10, DEFB3, HP, BPI, PYY2, AIF1, CD14, GRO1, CXCL2, IL18, IL1B, OLR1, CXCL2, DEFB1, IL1RN, ISG15, IFNAR1, IFNAR2, NFKBIZ, DEFB4A, CCL4, NOD2, FCN1, CFD, POLR3C, CCR7, NRROS, NLRC4, HMOX1, HYAL1, PTAFR, F2R, VNN1, S100A9, BNIP3L, NLRP3, PNMA1, LY96, AKIRIN2, IRF5, AFAP1L2, S100A8, ATG12, LYZ, IRF1*

Table S6. List of genes enriched under sub-category, immune response to stress in Sahiwal cows

Function	GO ID	Genes
Innate immune response	GO:0045087	*AIF1, CD14, ISG15, IFNAR2, NOD2, FCN1, CFD, POLR3C, NRROS, NLRC4, VNN1, S100A9, LY96, AKIRIN2, IRF5, S100A8, ATG12, IRF1*
Adaptive immune response	GO:0002250	*IL18, FAS, CTSS, NOD2, MSH2,EXOSC3, ERCC1*
Leukocyte activation involved in immune response	GO:0002366	*CX3CR1, PSEN1, MSH2, EXOSC3, ERCC1*
Lymphocyte activation involved in immune response	GO:0002285	*PSEN1, MSH2, EXOSC3, ERCC1*
T cell activation involved in immune response	GO:0002286	*CX3CR1, PSEN1, MSH2, EXOSC3, ERCC1*

Table S7. List of genes identified for sub-category, catalytic activity in Sahiwal cows

Function	GO ID	Genes
Oxidoreductase activity	GO:0016491	*COX7B, LDHA, CYP11B1, PRDX2, COX7A2, NDUFA4, ND1, ND2, ND4L, ND4, ND5, CYTB, NDUFA7, NDUFS7, GFOD2, LTC4S, PYCRL, ACOX1, SDR42E1, OXR1, PYCR1, IDH3G*
Transferase activity	GO:0016787	*MET, HAS2, LRAT, PIK3CA, FUT4, UBA1, COL4A3BP, GSTM1, ST3GAL5, ST6GALNAC6, ZDHHC16, PFKM, PFKL, ZNRF1, DPM3, LTC4S, PHKG2, ZDHHC6, METTL17, B4GALT3, RABGGTA, AK4, UBE4A, CDK2, TGFB2, RPN2, SUMO3, PRPS1*
Hydrolase activity	GO:0016740	*F9, DPP4, GNB2, MMP1, NT5E, ABCC1,IMPA1, MYO10, PCSK2, PDE1A, PLCB4, UCHL5, ENTPD1, MAN2B1, UQCRC2, RAB27B, PAG2, ATP5D, PPAP2C, SMPD1, KIF22, GNL3L, CPB2, DOM3Z, PNPLA2, ACAP1, CTDNEP1, ABHD4, ABHD1, SLX1A, HAGHL, RNASEK, GTPBP1, USP1,CDC25A, LPPR2, PSPH, LHPP, CLPP, CPPED1, PNKD, TMEM55A, PTRHD1, ATP5E, F7, PHPT1, KIF2A, ABHD8, PLCXD3, MYH2*
Nucleic acid binding transcription factor activity	GO:0001071	*FOS, ETS2, USF1, ATF4, TSC22D1, MYC, FOXP1, PHB, PKNOX1, TFDP1, CREBL2, CTNNB1, CERS2, HES1, NFYA, IRF5, DMRT1, GATAD1, IRF1*
Transmembrane transporter activity	GO:0022857	*FGF2, ITPR3, ATP1A1, ATP6V1A, COX6A1, SLC2A1, SLC2A3, SLC6A6, PSEN1, SLC25A22, KCNK1, NIPA2, SLC25A29, SFXN4, SLC38A7, SFXN2, CLCN7, UQCRH, SLC39A13, TTYH1*

Table S8. List of significantly enriched pathways in Ladakhi and Sahiwal cows PBMCs

Pathways	p Value	Matched entities	Pathway entities of experiment type	Up-regulated genes in Ladakhi cows	Down-regulated genes in Ladakhi cows
MAPK_signaling_pathway_WP998_48136	5.85E-04	39	155	14	25
Cell_Receptor_Signaling_Pathway_WP1025_40541	0.00228197	35	150	07	28
TGF- beta_Receptor_Signaling_Pathway_WP1045_40538	0.00200879	35	146	14	21
Insulin_Signaling_WP966_48134	0.05163705	30	153	12	18

T_Cell_Receptor_Signaling_Pathway_WP1011_40515	0.00466109	30	128	14	16
TNF-alpha_NF-kB_Signaling_Pathway_ WP1047_40552	0.44205162	27	178	7	20
Myometrial_Relaxation_and_Contraction_Pathways_WP3276_80929	0.15414445	25	151	10	15
Regulation_of_toll-like_ receptor_signaling_pathway_WP3132_80689	0.032885857	25	124	6	19
mRNA_Processing_WP1023_80888	0.08064093	23	124	8	15
Apoptosis_WP1018_81196	0.00457543	20	79	7	13
Senescence_and_Autophagy_WP1058_48391	0.0500804	20	90	9	11
Vascular_smooth_muscle_contraction_WP2912_80970	0.3755309	19	122	10	9
B_Cell_Receptor_Signaling_Pathway_WP1025_80807	0.04917480	18	94	4	14
Toll-like_ receptor_ signaling_ pathway_ WP1067_40560	0.12496923	18	92	4	14
Electron_Transport_Chain_WP1002_80879	0.10293593	17	100	12	5
G_Protein_Signaling_Pathways_WP1049_40582	0.1324902	16	89	8	8
DNA_Damage_Response_WP3143_80705	0.24667534	16	104	5	11
Delta-Notch_Signaling_Pathway_WP1064_ 40530	0.09930001	16	78	7	9
IL-2_Signaling_Pathway_WP973_40493	0.06653483	16	73	7	9
IL-3_Signaling_Pathway_WP1031_40554	0.39197975	16	98	4	12
IL-6_Signaling_Pathway_WP976_40574	0.34170285	16	96	3	13
TSH_signaling_pathway_WP3227_80831	0.00541485	16	64	5	11
Apoptosis_Modulation_and_Signaling_WP3148_80711	0.1373349	15	81	5	10

Table S9. Gene symbol, amplicon size, slope, PCR efficiency and R^2 of internal control genes

Gene Symbol	Accession Number	Primers 5'-3' (Forward, Reverse)	T_a(℃)	Amplicon Size (bp)
ACTB	AY141970	F:5'GCGTGGCTACAGCTTCACC3'<br>R:3'TTGATGTCACGGACGATTTC5'	60	56
GAPDH	BC102589	F:5'TGGAAAGGCCATCACCATCT3'<br>R:3'CCCACTTGATGTTGGCAG5'	60	60
EEF1A1	BC105315	F:5'CATCCCAGGCTGACTGTGC3'<br>R:3'TGTAAGCCAAAAGGGCATGC5'	60	101
B2M	NM_173893	F:5'CTGCTATGTGTATGGGTTCC3'<br>R:3'GGAGTGAACTCAGCGTG5'	60	101
HMBS	BC112573.1	F:5'CTTTGGAGAGGAATGAAGTG3'<br>R:3'AATGGTGAAGCCAGGAGGAA5'	60	80
RPL4	NM_001014894	F:5'TTGGAAACATGTGTCGTGGG3'<br>R:3'GCAGATGGCGTATCGCTTCT5'	60	101
RPS15	BC108231	F:5'GAATGGTGCGCATGAATGTC3'<br>R:3'GACTTTGGAGCACGGCCTAA5'	60	101
RPS23	BC102049	F:5'CCCAATGATGGTTGCTTGAA3'<br>R:3'CGGACTCCAGGAATGTCACC5'	60	101
RPS9	DT860044	F:5'CCTCGACCAAGAGCTGAAG3'<br>R:3'CCTCCAGACCTCACGTTTGTT5'	60	54
UXT	CR452243	F:5'TGTGGCCCTTGGATATGGTT3'<br>R:3'GGTTGTCGCTGAGCTCTGTG5'	60	101
HPRT1	BC103	F:5'GAGAAGTCCGAGTTGAGTTT3'<br>R:3'GGCTCGTAGTGCAAATGAAG5'	60	101

Table S10. Gene symbol, Gene Bank accession numbers, primer sequences and annealing temperature (Ta) of hypoxia related target genes

Gene Symbol	Description	Amplicon size (bp)	PCR efficiency (%)	Slope	R^2 Value
HIF-1α	F:5'TGAAGGCACAGATGAATT3'<br>R:3'GTTCAAACTGAGTTAATCC5'	129	102	-3.26	0.985
VEGFA	F:5'TTCCAGGAGTACCCAGATG3'<br>R:3'CTGGCTTTGGTGAGGTTTG5'	162	103	-3.24	0.997
SLC2A1	F:5'TCCACAAGCATCTTCGAGA3'<br>R:3'AATAGCGACACGACAGTGA5'	98	96	-3.42	0.999
HK2	F:5'AAGATGCTGCCCACCTACG3'<br>R:3'TCGCTTCCCATTCCTCACA5'	123	102	-3.26	0.992
GRα	F:5'CCATTTCTGTTCACGGTGTG3'<br>R:3'CTGAACCGACAGGAATTGG5'	132	111	-3.08	0.997
TNFα	F:5'AGGTGGCCCCTCCATCA3'<br>R:3'GGCTACCGGCTTGTTACTT5'	79	121	-2.89	0.972
EPAS-1	F:5'AGCAAGCCTTCCAAGACAT3'<br>R:3'GCTTGTCCGGCATCAAAGA5'	90	115	-3.00	0.990
NOS2	F:5'ACGAGGAACAGGTGGAGG3'<br>R:3'TGCTGCCCCATCCTTTTTC5'	110	107	-3.25	0.980
ECE1	F:5'GCCTACTACTCGCCCACCA3'<br>R:3'CGCCGAAGTTTAAGGCATT5'	100	122	-2.88	0.988
HSP27	F: 5'TACATTTCCCGTTGCTTCA3'<br>R: 3'GGACAGAGAGGAGGAGAC5'	78	103	-3.26	0.985
HSP70	F:5'AACATGAAGAGCGCCGTGG3'<br>R:3'GTTACACACCTGCTCCAGC5'	171	121	-2.89	0.994
HSP90	F:5'ACATGCCAACAGGATCTAC3'<br>R:3'CTGTCATCAGCAGTGGG5'	74	89	-3.60	0.998
